# Supplementary material for: Chemical staining for fundamental studies and optimization of binders in Li-ion battery negative electrodes
Source: Nat Commun. 2026 Feb 17;17:1438. doi: 10.1038/s41467-026-69002-1 (PMC12913984; doi:10.1038/s41467-026-69002-1)
Supplement: Supplementary file 1 — Supplementary Information [file 41467_2026_69002_MOESM1_ESM.pdf]

Supplementary Information

Chemical staining for fundamental studies and optimization of binders in Li-ion battery negative electrodes

Stanislaw P. Zankowski[1,2,], Samuel Wheeler[1,2], Thomas Barthelay[1,2], Wai Man Chan[3], Michael Metzler[1], Patrick S. Grant[1,2,**]*

1. Department of Materials, University of Oxford, Parks Road, Oxford OX1 3PH, United Kingdom

2. The Faraday Institution, Quad One, Harwell Campus, OX11 0RA, United Kingdom

3. Oxford Materials Characterisation Service, Department of Materials, University of Oxford, Begbroke Science Park, Oxford OX5 1PF, United Kingdom

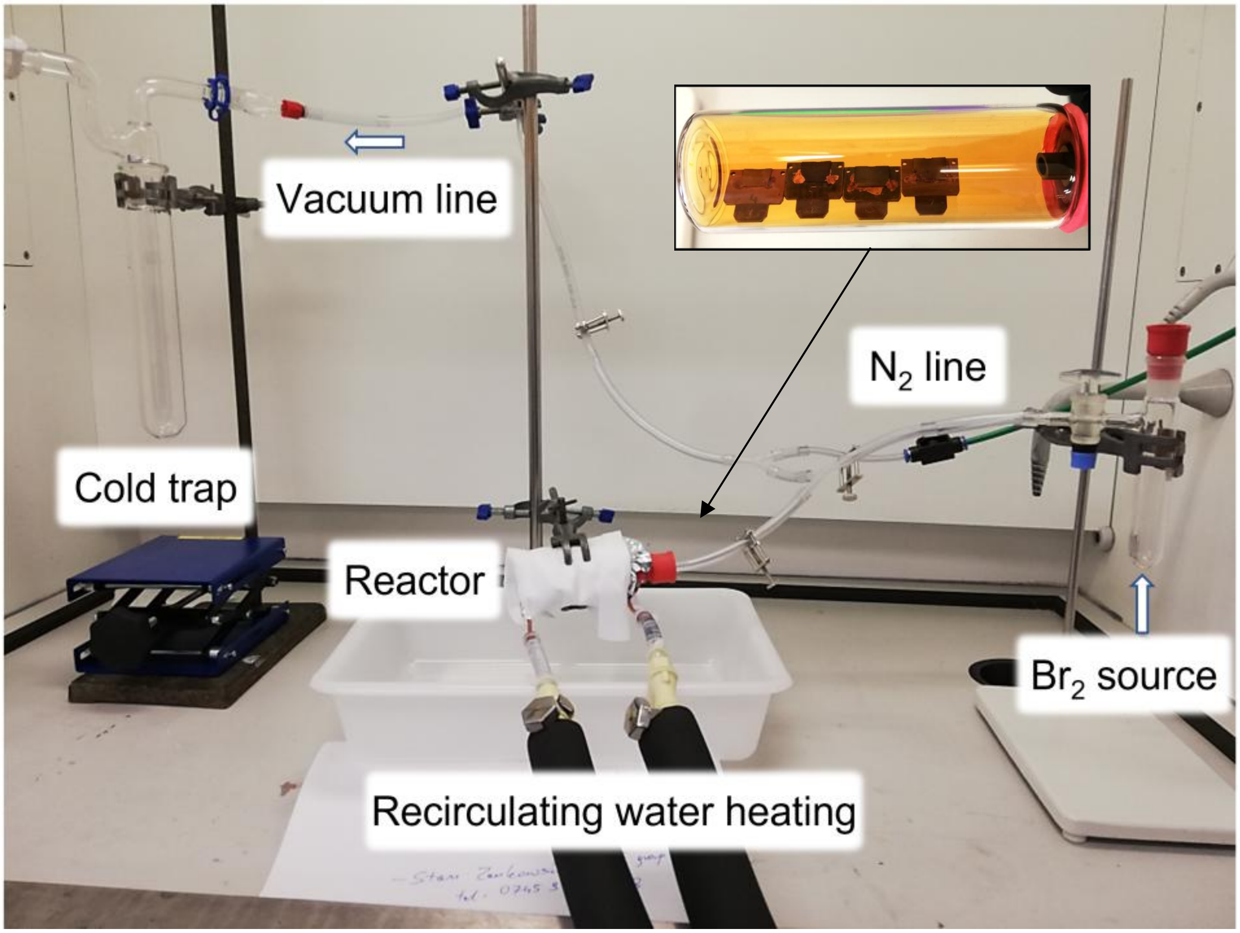

Figure S1. Photograph of the bromination setup, together with an inset showing the glass reactor during bromination of electrode samples mounted on PECS sample holders.

Supplementary Note S1. Mechanism of the staining reactions

The mechanism of staining reactions was first assessed by attenuated total reflection infrared spectroscopy (ATR-IR) and energy-dispersive X-ray spectroscopy (EDX) (Figure S2). Fig. S2A shows ATR-IR spectra of pristine and freshly brominated SBR. As expected, the spectra of SBR before and after bromination indicate diminishing intensity of aliphatic C=C peaks, particularly the trans peak at 967 cm^{-1}, vinyl peaks at 909, 995 and 1640 cm^{-1} and cis peak at 1403 cm^{-1}, with the appearance of a broad shoulder around 1225 cm^{-1} which could be ascribed to the C-H wagging vibrations within the CH_2-Br group.[1–3] Additionally, the appearance of the carbonyl peak at 1717

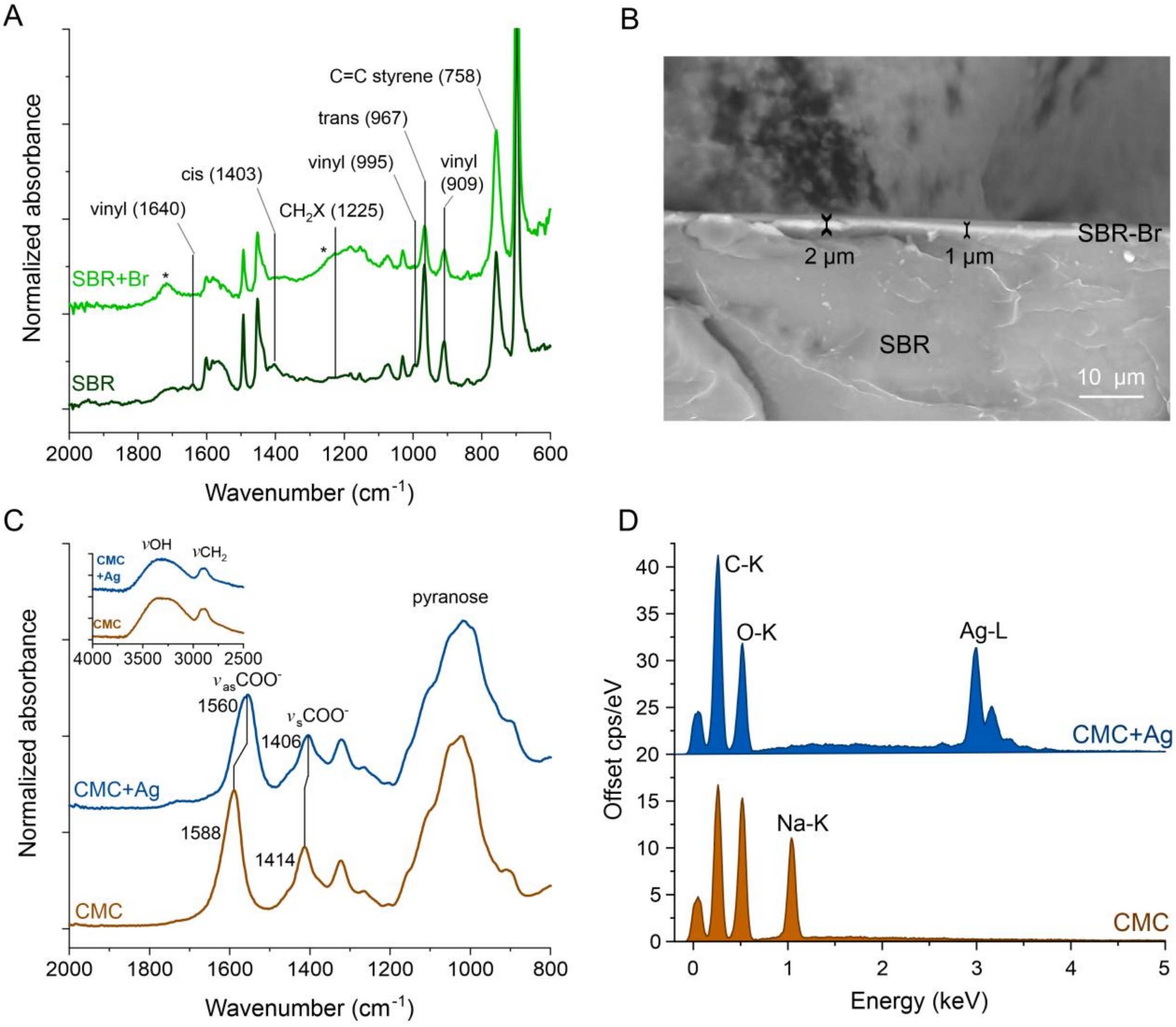

Figure S2. A) ATR-IR spectra of SBR before and after bromination. The asterisks indicate peaks due to by-products of C-Br hydrolysis, such as carbonyl and ester groups. B) Backscattered electron image of a cross section of SBR film after bromination, showing the top part of the film that was exposed to Br_2, where the visible bright layer indicates the depth of brominated SBR. C) ATR-IR spectra of Na-CMC before and after staining with Ag^+. The inset shows the OH/CH_2 spectral region. D) EDX spectra of Na-CMC before and after staining with Ag^+, showing disappearance of Na-K peak in the sample upon silverization.

cm^{-1} and, potentially, ester peak at 1240 cm^{-1} can be indicative of the partial decomposition of C-Br bonds in air.[4] This reaction does not occur at high enough rate to be of concern for EDX mapping of binders, as we did not observe significant lowering of Br concentration in samples exposed to air for up to a year. In the tested conditions, bromination of a thick and compact SBR

film resulted in bromination of the top 1-2 μm part of the film, as evident from the backscattered electron image of the sample cross section shown in Fig. S2B.

Fig. S2C shows ATR-IR spectra of Na-CMC before and after silver staining. The spectra show four distinctive regions: 3700-3000 cm^{-1} related to vibrations of OH groups (linked to pyranose rings of CMC, physiosorbed water and intra- and intermolecular hydrogen bonds), 3000-2800 cm^{-1} related to vibrations of CH and CH_2 groups, 1800-1200 cm^{-1} related to vibrations of carboxylate groups and < 1200 cm^{-1} related to vibrations of pyranose rings and glycosylic linkages between them.[5–7] The analysis of the spectra reveals strong interactions between the complexing metal ions and carboxylate groups of CMC upon staining with Ag^+, as evident by the downshifting of the peaks at 1588 cm^{-1} and 1414 cm^{-1} associated with asymmetric and symmetric vibrations of COO^- groups. The separation between these two peaks can be also related to the structure of the carboxylate-metal complex.[8,9] The peak separation of 174 ± 2 cm^{-1} for Na-CMC and 154 ± 4 cm^{-1} for Ag-CMC indicate that Ag binding changes the metal-CMC coordination from bidentate bridging to bidentate chelating geometry[8,9] (note, here and thereafter, ± indicates 95% confidence intervals of the average values). Although there were no significant changes in the hydroxyl region between the samples, the proper analysis of this region may be obstructed by the presence of adsorbed water. Additionally, the EDX spectra of the CMC film before and after silver staining presented in Fig. S2D show that silverization of CMC occurs via substitution of Ag for Na, as evident by the disappearence of Na-K line at 1.04 keV and appearance of Ag-L multiplet at 2.98 keV in the silverized CMC. This also shows that silverization is complete within at least the upper 1-2 microns of the CMC film, which is the estimated depth of EDX Ag-L probing in the CMC film at 15 kV of electron beam voltage used in this analysis.

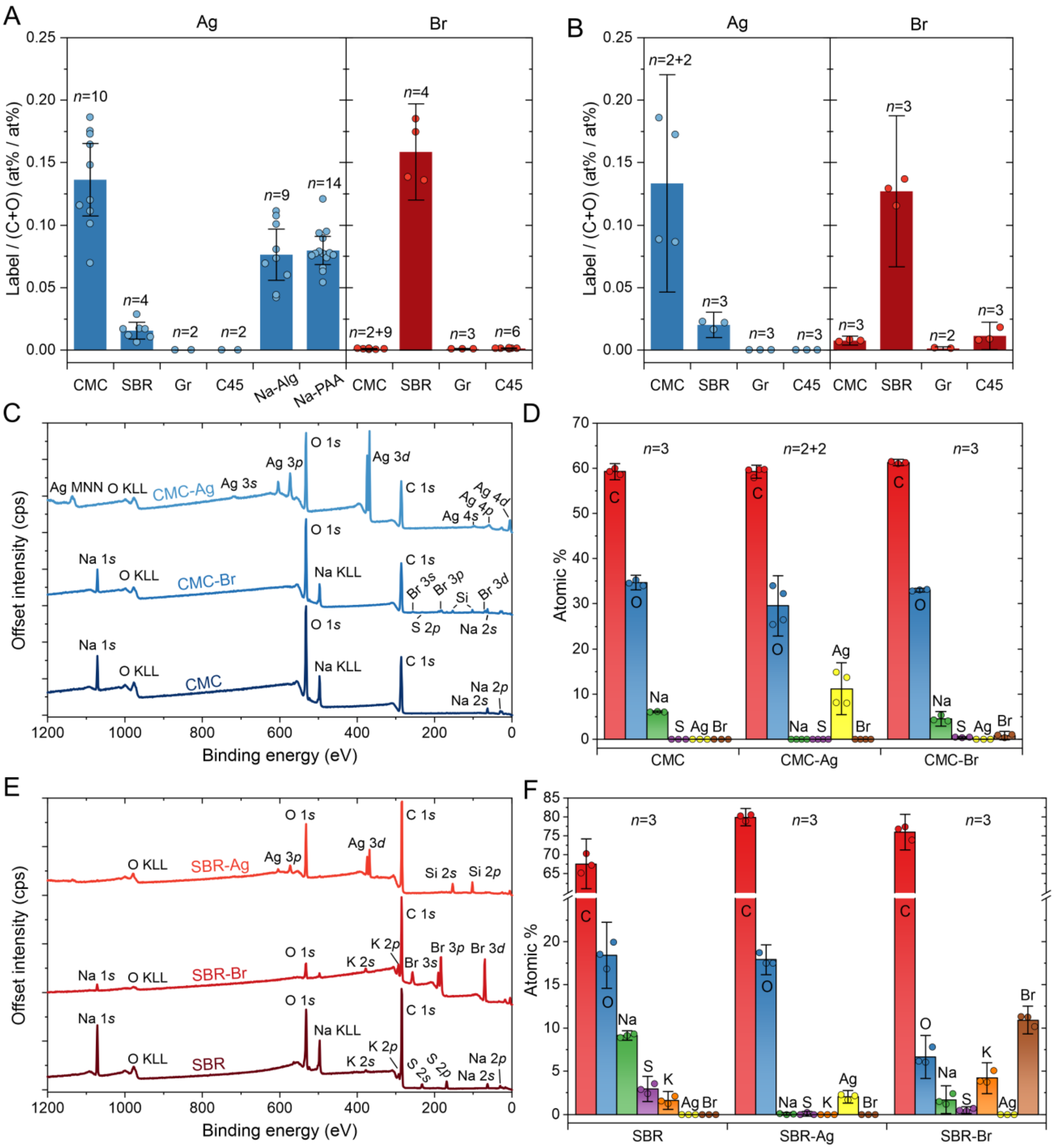

Figure S3. A) Mean relative amount of Ag and Br (labels) in films of CMC and SBR and powders of graphite (Gr) and C45 carbon conductive additive after silverization and bromination, determined with EDX. The figure also shows quantification of Ag in silverized sodium alginate (Na-Alg) and sodium polyacrylate (Na-PAA). B) Mean relative amount of Ag and Br (labels) in films of CMC and SBR and powders of graphite (Gr) and C45 carbon conductive additive after silverization and bromination, determined with XPS. C) XPS spectra and D) mean XPS elemental composition of CMC before and after bromination and silverization. E) XPS spectra and F) mean XPS elemental composition of SBR before and after bromination and silverization. The error bars in A), B), D), F) correspond to 95% confidence intervals of averages. In A) and B), the confidence intervals also include systematic quantification uncertainty – 5.2% and 4.2% relative for Br and Ag with EDX, respectively, and 20% relative assumed for XPS. In A), B), D), F) the number of technical replicates is annotated as *n* (note that XPS of CMC-Ag and EDX of CMC-Br was additionally performed on two biological replicates in each case). Note that the elemental quantification in D) and F) excludes Si found in CMC-Br, SBR-Br and SBR-Ag (1.8 at%, 0.5 at% and 9.0 at%, respectively). EDX was acquired at 15 kV beam voltage.

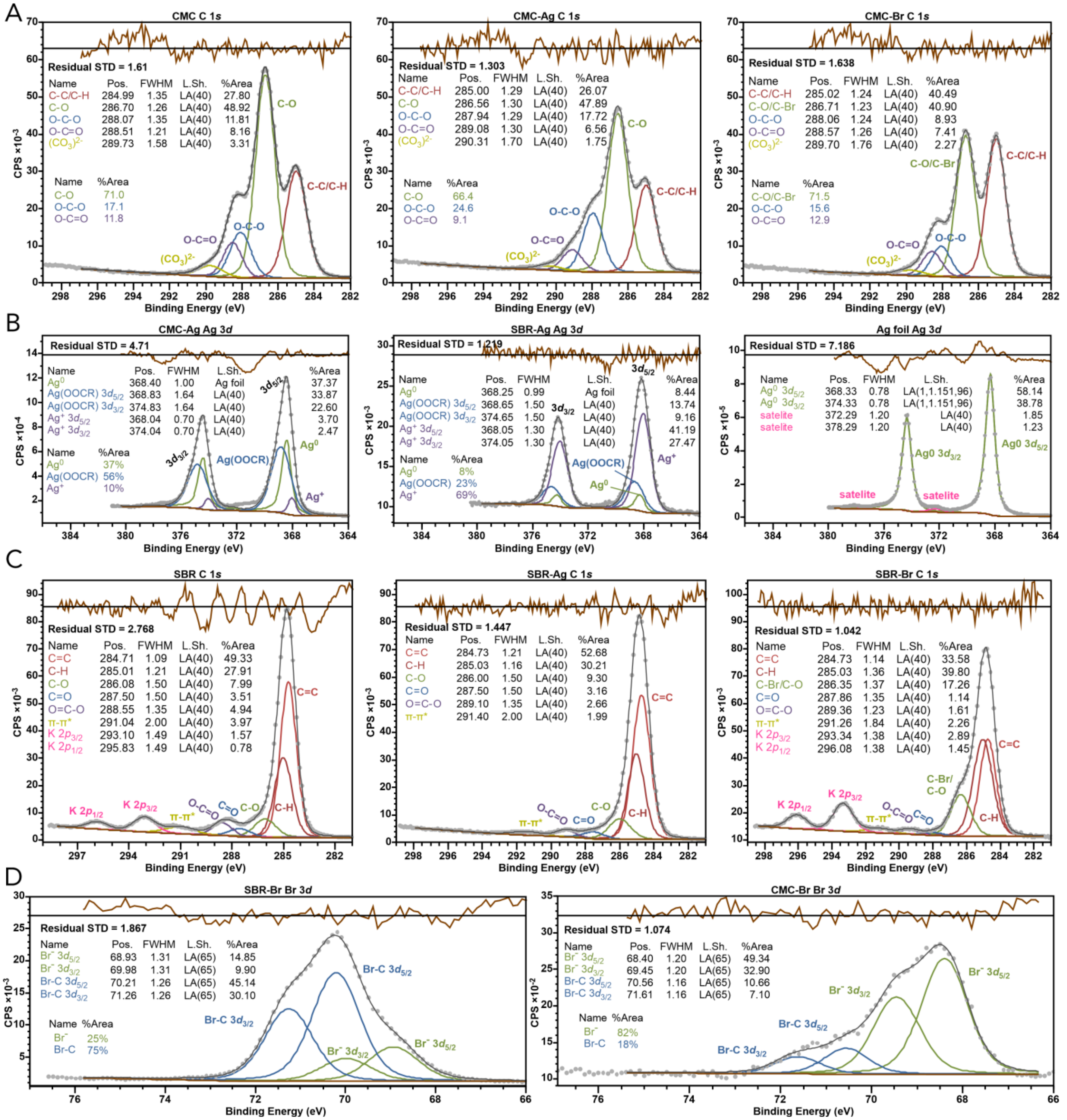

Figure S4. XPS core region analysis of CMC and SBR before and after silverization and bromination. A) C 1*s* region of CMC, silverized CMC and brominated CMC; B) Ag 3*d* region of silverized CMC and SBR, together with a spectrum of metallic Ag foil used to create empirical Ag0 peak model (the results of fitting Ag foil with asymmetric peak shapes are shown for informative purpose only); C) C 1*s* analysis of SBR, brominated SBR and silverized SBR; D) Br 3*d* region of brominated SBR and brominated CMC. For each sample, binding energy was corrected by referencing to the fitted C 1*s* C-C/C-H peak at 285.0 eV. Dark grey lines represent summed fitting envelopes. Note that due to close proximity of some of the peaks (e.g. C=C and C-H, O-C=O and O-C-O, Ag^0 and Ag^{+}), the peak models and quantitative analysis of C 1*s* and Ag 3*d* regions have a significant level of uncertainty. The C 1*s* and Br 3*d* spectra were processed with the universal polymer Tougaard background and the Ag 3*d* spectra with the iterated Shirley background that stabilized the Ag 3*d* fitting model.

Supplementary Note S2. XPS analysis of CMC and SBR staining

To elucidate further on the staining reactions mechanism and selectivity, we performed X-ray photoemission spectroscopy (XPS) on raw and stained films of CMC and SBR. Figures S3C and S3D present survey spectra and elemental analysis of CMC before and after the staining. Elemental analysis of raw CMC yielded 6.0±0.2 at.% Na, 34.7±1.6 at.% O and 59.3±1.8 at. % C. Na-CMC having degree of substitution (DS) of x can be described using a stoichiometry of $C_{6+2x}O_{5+2x}Na_x$ (excluding hydrogen). Thus, DS can be estimated based on Na at% or Na/O at.% ratio y according to equations (S1) and (S2), respectively:

$$x_{\mathrm{Na}} = 11\frac{\mathrm{Na}}{100 - 5\mathrm{Na}} = 0.95 \tag{S1}$$

$$x_{\mathrm{Na/O}} = -5\frac{y}{2y - 1} = 1.33 \tag{S2}$$

While the x_{Na} is in good agreement with the DS of the CMC used in our work (0.8-0.95), the $x_{\mathrm{Na/O}}$ is overestimated by at least 40%. This could be related to 9.1 at.% oxygen deficiency in the material compared to the theoretical 43.8% O in Na-CMC with DS = 0.95, which is also consistent with the 9.1 at.% excess of C detected in our samples compared to the nominal 50.2% C for Na-CMC with DS = 0.95. This indicates that CMC could be partially degraded during XPS analysis, which is consistent with the reductive degradation behavior of analogous sodium alginate during XPS acquisitions,[10] and is indicative of an increased uncertainty in interpreting the XPS results. Following silverization, the Ag-CMC showed 11.2±5.8 at% Ag, an unchanged C content of 59.3±1.5 at%, and the decrease of O content to 29.6±6.7 at%. The decrease of O content could indicate partial decomposition of the oxygenated functional groups by the Ag^+ which could become reduced to Ag^0, as previously reported for the CMC complexed with Ag^+.[11,12] After bromination, the composition of CMC remained largely unchanged, with a small amount of Br (0.7±0.9 at%), S (0.4±0.2 at%) and Si (1.9±0.4 at%). The presence of Si and S could be due to

residual contamination with silicon oil from the Schlenk apparatus used during bromination and slight cross-contamination of the CMC sample with S from the SBR stained in the same reactor, as described below.

Figure S3D and S3E shows survey spectra and elemental analysis of SBR before and after staining. Besides 67.6±6.6 at% C in raw SBR film, the sample also contained large amount of O (18.4±3.8 at%), Na (9.1±0.6 at%), S (2.9±1.5 at%) and K (1.6±1.0 at%). The detection of these elements, also separately confirmed with EDX, is likely due to the emulsifiers (e.g. sodium dodecyl sulphate) and SBR polymerization byproducts present within the SBR samples, as discussed in more detail in Supplementary Note S3 below. Silverization of SBR resulted in binding of some Ag (2.0±0.8 at%) and disappearance of Na, S and K, which could be due to substitution of these elements with Ag^{+} during silverization and their subsequent removal during sample washing in water. Note, however, that this sample also showed about 9.0±1.5 at.% of Si, possibly from the cut glass substrate residuals used to support the SBR film, or another unknown source of contamination. In the brominated SBR, 10.9±1.5 at% of Br was detected together with a lower O, Na and S content of 6.0-7.8 at% O, 1.2-2.5 at% Na and 0.4-0.7 at% S, respectively, and an increased K content of 3.8-5.0 at%. These changes compared to the unstained SBR could be either due to the degradation and removal of some of the SBR emulsifiers/polymerization byproducts during bromination and subsequent blowing with N_2, but could also result from the random migration of these elements during SBR film cross-linking and drying.

Detailed chemical analysis of the staining reactions was performed using high-resolution C 1*s*, Ag 3*d* and Br 3*d* spectra of the CMC and SBR samples before and after the staining presented in Figure S4. Fig. S4A shows C 1*s* region of CMC before and after staining, fitted with symmetric Voigt-shaped peaks in the binding energy intervals of 286-287 eV (C-O/C-OH carbons), 287.7-

288.1 eV (O-C-O carbon) and 288.2-289.5 eV (carbon in COO^- anion).[13] In the dried CMC film, the fitted binding energy of carboxylate anion at 288.5 eV was slightly lower than the typical energy of carboxylic acid group (~290.0 eV), consistent with the electron delocalization in deprotonated COO^- anion.[14] The C 1*s* spectra of CMC samples additionally revealed a large peak at 285.0 eV (aliphatic C-C/C-H) and a minor broad peak at 289.8 eV assigned to the carbonate group, originating either from surface contamination or partial X-ray-induced decomposition of the CMC surface – in agreement with the previous survey elemental analysis. We should note that the presence of C-C/C-H peak at 285.0 eV is frequently reported in the C 1*s* spectra of Na-CMC and similar polysaccharides[10,11,15–18], but is often mistakenly assigned to C-C ring carbons within the CMC structure (pure CMC should not exhibit such peak, having all carbons bound to at least one oxygen). To verify the accuracy of the fitted peak model, we considered the CMC spectrum – after excluding the contribution of C-C/C-H and CO_3^{2-} peaks, we estimated the relative peak areas of C-O, O-C-O and O=C-O groups as 71.0%, 17.1% and 11.8%, respectively. Considering that the C-O peak is a sum of contributions of the pyranose C-O carbons and the CH_2O carbon of the carboxymethyl substituent (the latter with the same peak area as the COO^- peak), the cellulose C-O peak area was calculated as 71.0% - 17.1% = 59.2%, giving the ratio of cellulose C-O to O-C-O peak areas of 3.5:1, which is 30% less than the theoretical 5:1 ratio. Although the lower-than-theoretical ratio of these functional groups could be indicative to the X-ray induced CMC dehydroxylation (as proposed by Kabachkov et al. for the analogous sodium alginate[10]), which is also supported by our evidence of CMC degradation from the survey spectra analysis discussed earlier, it could also originate from some inaccuracy of our peak model. Additionally, we estimated the degree of substitution of Na-CMC based on its stoichiometry using the relation provided in equation (S3):

$$\mathrm{DS} = 6 * A_{\mathrm{COO^-}} * (100\% - 2 * A_{\mathrm{COO^-}})^{-1} \quad \text{(S3)}$$

where the DS is the degree of substitution and A_{COO^-} is the % of carboxylate peak area in the sum of carboxylate, C-O and O-C-O peak areas), giving DS of 0.93, which is within 2% of the DS = 0.95 estimated earlier from the Na atomic concentration, and within 2-16% of the nominal DS of the Na-CMC used in our experiments (0.8 – 0.95). Combined, the 2-30% uncertainty of the C 1*s* peak model is due to the factors such as close proximity of the peaks (including peak overlap of O-C-O and C=O groups – the latter could originate from sample decomposition or contamination), peak broadening (as an effect of e.g. polymer cross-linking in the dried films), uncertainty of exact peak shapes, possible polymer degradation (indicated by the CO_3^{2-} and C-C components), potential peak asymmetry (not modeled here), uncertainty of binding energy correction to the C-H peak position of 285 eV and uncertainty related to the background fitting.[13,19,20]

After silverization of CMC, the overall intensity of the C 1*s* region in Fig. S4A decreased, indicating some decomposition of the CMC surface. The carboxylate anion peak was shifted to a higher binding energy of 289.1 eV, indicating change in chemical environment of the COO^- group – in agreement with the ATR data described previously in Supplementary Note S1. Also, the relative areas of the COO^- and C-O peaks decreased to 66.4% and 9.1%, with an increase of the O-C-O area to 17.7%. This could be due to reduction of the C-OH and COO^- groups by the complexed Ag^+ and formation of metallic Ag^0 nanoclusters,[12] in agreement with the survey elemental analysis in Fig. S3. This was also supported by the analysis of Ag 3*d* region of this sample (Fig. S4B), where an empirical asymmetric Ag^0 peak model synthesized from separately measured Ag foil (also shown in Fig S4B) with the $3d_{5/2}$ binding energy of 368.3-368.4 eV was fitted next to a broader symmetric doublet with a higher $3d_{5/2}$ binding energy of 368.8 eV (assigned to Ag-carboxylate complex) and a doublet at a lower $3d_{5/2}$ binding energy of 368.0 eV, assigned

to not-complexed Ag^+.[21–23] In SBR-Ag, the Ag 3*d* region was primarily composed of the lower-binding energy Ag^+ ionic component, with a smaller contribution of Ag^0 and Ag-carboxylate peaks. This could be explained by Ag^+ binding primarily to the anionic groups (e.g. SO_4^{2-} in sodium dodecyl sulphate) and, to a lower degree, fatty acid groups of the SBR emulsifiers (again, see Supplementary Note S3 for the discussion on emulsifiers and polymerization by-products found in SBR, also showing evidence of SO_4^{2-} found in SBR).[21] We should note, however, that the close proximity of Ag peaks together with sensitivity to binding energy correction and the asymmetry of Ag^0 peaks makes accurate distinction of Ag oxidation states in the Ag 3*d* region difficult.[21] Also, the measured Ag 3*d* spectra had complex shapes with high binding energy tails leaving large fitting residuals (particularly visible in the CMC-Ag spectrum), which could be due to the complex chemical environment of the Ag-carboxylate complex influenced by the hydroxyl groups of the adjacent CMC chains.

In brominated CMC, the C 1*s* region showed no significant differences in the relative C-O, O-C-O and COO^- peak area ratios, but there was a marked increase in the C-C/C-H peak area at 285 eV. The spectrum of this sample also showed an overall decrease of C 1*s* region intensity, indicating partial decomposition of the material. We should note that the potential presence of residual silicon oil contamination from the Schlenk apparatus, as described earlier, may have partially contributed to the increased C-C/C-H peak area in this sample.

The C 1*s* region of dried SBR film in Fig. S4A was dominated by the signal at 284.5-285 eV, which was fitted with two peaks separated by 0.3 eV and assigned to C=C carbons at 284.7 eV and C-H carbon at 285.0 eV (due to the unsaturated C=C, a π-π* shakeup satellite was also visible at 291.2 eV). There were also small peaks at higher binding energy due to oxidized carbon groups, together with a K 2*p* doublet at 293.1 and 295.8 eV due to the SBR emulsifiers and by-products

of polymerization. The larger amount of C=C compared to aliphatic C-C/C-H is consistent with the structure of SBR, where the unsaturated aliphatic and aromatic carbons are in excess to the saturated sp^3 carbons. In silverized SBR, the C 1*s* spectrum showed disappearance of K 2*p* doublet due to its potential substitution with Ag^+ and removal during the post-silverization water rinse.

In brominated SBR, there was a marked increase of the signal at 286.3 eV due to the presence of C-Br groups (note, the position of C-Br peak overlaps with that of the C-O peak[13,24]), together with the decrease of the unsaturated C=C peak intensity. Furthermore, analysis of the Br 3*d* region (Fig. S4D) allowed to denconvolute two chemical states of Br by fitting two pairs of doublets with 1.05 eV peak-to-peak separation: Br^- and Br-C having the larger $3d_{5/2}$ peak centered at 68.6-69.1 eV and at 70.1-71.1 eV, respectively.[23,25] Thus, about 75% of Br in brominated SBR was found bounded to carbon in the polymer structure, with the remaining 25% Br present as a Br^- anion. This is opposed to Br in brominated CMC where 82% of Br was found to exist as Br^- anion, which is consistent with the formation of NaBr in this sample – as also suggested by the elemental EDX quantification presented in Figure S7.

Supplementary Note S3. Selectivity of silverization in the top- and bulk regions of electrodes.

To analyse selectivity of Ag binding to CMC over SBR in the top and bulk regions of graphitic electrodes, we tested it with EDX and energy-selective backscattered electron imaging (EsB) on slurry-cast electrodes containing graphite, 1 wt.% C45 and only one of the binders – either 1.5 wt.% CMC or 2.3 wt.% SBR. These specific formulations were used to reflect the binder mix (1.5 wt.% CMC + 2.3 wt.% SBR) in our typical slurry-cast electrodes analyzed throughtout this study, following the industry practices and standard electrode formulation adapted across the UK's Nextrode consortium.[26] Measurements of the top electrode surface were taken directly on the top

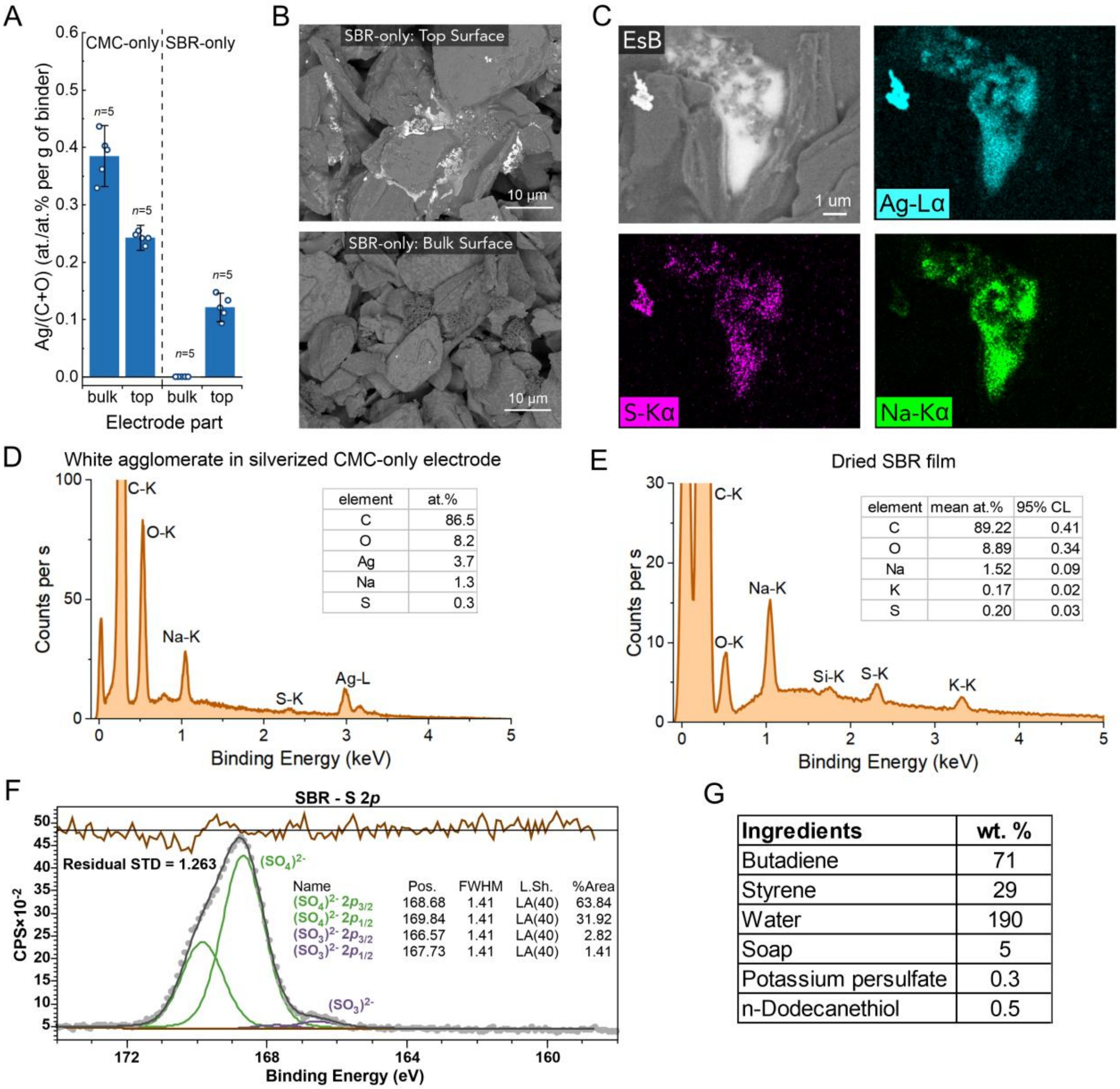

Ingredients	wt. %
Butadiene	71
Styrene	29
Water	190
Soap	5
Potassium persulfate	0.3
n-Dodecanethiol	0.5

Figure S5. A) Mean relative Ag amount in top and bulk surfaces of silverized single-binder electrodes, based on EDX analyses of *n*=5 random areas for each electrode fragment. The error bars represent 95% confidence intervals of the averages. B) Energy-selective backscattered electron (EsB) images of the top and bulk surfaces of silverized SBR-only electrode, showing association of silver (white contrast) in particulate and amorphous agglomerates between graphitic particles. C) EDX elemental mapping of one of the agglomerates appearing white in the EsB images, for Ag (teal), S (pink) and Na (green). D) EDX spectrum and elemental quantification of the agglomerate area shown in C). E) EDX elemental analysis of dried SBR film, acquired at 15 kV of beam voltage over *n*=20 different locations on the sample. The analysis is sensitive to about 1-2 microns of sample thickness. F) XPS S 2*p* core level spectrum of SBR, fitted with two 2*p* doublets for SO_4^{2-} (168.7 eV) and SO_3^{2-} (166.6 eV). Binding energy was referenced to the fitted C-H peak energy at 285.0 eV. G) An example of typical SBR emulsion polymerization recipes, taken from Ref.[27]

surface of the electrodes after silverization, while the analysis of the bulk region of the electrodes was done by first removing the top electrode surface using sticky carbon SEM discs, followed by standard silver staining and EDX/EsB analysis of the exposed internal (bulk) region of the electrode. Fig. S5A shows relative Ag amount quantified with EDX in the CMC- and SBR-only electrodes, together with EsB images of silverized SBR-only electrodes in Fig. S5B. EDX results in Fig. S5A show that silverization remained highly selective to CMC over SBR in the bulk of the electrodes, as indicated by the presence of Ag in the silverized CMC-only electrode and no detectable Ag in the bulk of the silverized SBR-only electrode. Surprisingly, a significant amount of Ag was detected at the top of the SBR-only electrode. This was consistent with the lack of visible bright Ag features in the EsB image of the internal SBR-only electrode's surface and the appearance of bright microparticles and white agglomerates in the image of the SBR-only electrode's top surface (Fig. S5B). EDX mapping shown in Fig. S5C revealed that these white agglomerates contained Ag, Na and S (the quantitative analysis of the agglomerate is presented in Fig. S5D). Na, S and K were also detected by EDX and XPS in the standalone dried SBR film prepared from the stock emulsion of SBR used in the preparation of graphitic electrodes (Fig. S5E and Fig. S3C, respectively). Considering that pure SBR should contain only C, O and H, the presence of Na, K and S in the dried SBR film and SBR-only electrodes showed that these elements must stem from the stock commercial SBR emulsion. Analysis of typical recipes used in emulsion-polymerization preparation of SBR (shown in Fig. S5G) suggests that these elements are part of leftover emulsion-polymerization agents, such as soap/emulsifiers (e.g. sodium lauryl sulfate), initiators (potassium persulfate) and chain transfer agents (thiols).[27] Presence of such compounds is also consistent with the XPS S 2*p* region analysis of dried SBR film in Fig S5F, showing predominance of sulphur in the SO_4^{2-} form.[28] The significantly larger quantity of Na, S and K

detected in the top 10 nm of the SBR film with XPS (e.g. 9 at. Na, 3 at.% S, 1 at.% K) compared to 1.5 at.% Na, 0.2 at.% S and 0.2 at.% K detected in the first 1 μm with EDX shows that these compounds tend to migrate to the top of the dried samples. Because of the presence of carboxylate and sulphate groups, these compounds can also bind Ag^+ and progressively reduce it to Ag particles.[29] Together, this can explain the presence of both particulate and amorphous agglomerated Ag, and the fact that in the silverized SBR-only electrode, Ag was detected only on the top electrode surface (Fig. S5A).

Supplementary Note S4. The origin of bromine uptake in brominated CMC-only electrodes

Upon brominating a graphitic electrode containing 4% wt. of CMC as the only binder, a significant amount of Br was detected both within and on top of the electrode, and CMC could be observed with positive contrast in the BEI images, similarly to the appearance of Ag-CMC in silverized electrodes. High resolution EDX mapping revealed concentartion of Br in porous C45/CMC agglomerates, shown in Fig. S6A. Elemental analysis of these agglomerates shown in Fig. S6B revealed a 1:1 atomic ratio of Br to Na, suggesting formation of NaBr. On the other hand, XPS analysis of brominated standalone CMC film shows a much lower Br:Na ratio of 1:10 (Fig. S6C). Considering the differences in the analysis depth of EDX and XPS, but also the siginificantly lower surface-to-volume ratio of CMC in compact CMC film compared to the CMC in porous CMC/C45 agglomerate with extended surface area, the differences in Br:Na detected by XPS and EDX indicate highly surface-limited character of CMC bromination. Because of the highly oxidative nature of Br_2, the reaction may involve oxidation of e.g. carboxylate groups within the CMC structure, reduction of Br_2 to Br^- and its binding to Na associated with the carboxylate groups in Na-CMC.[30] This reaction and formation of NaBr is consistent with the mostly ionic character

of Br found with XPS Br 3*d* analysis in brominated CMC, as discussed earlier in Supplementary Note S2 and Figure S4.

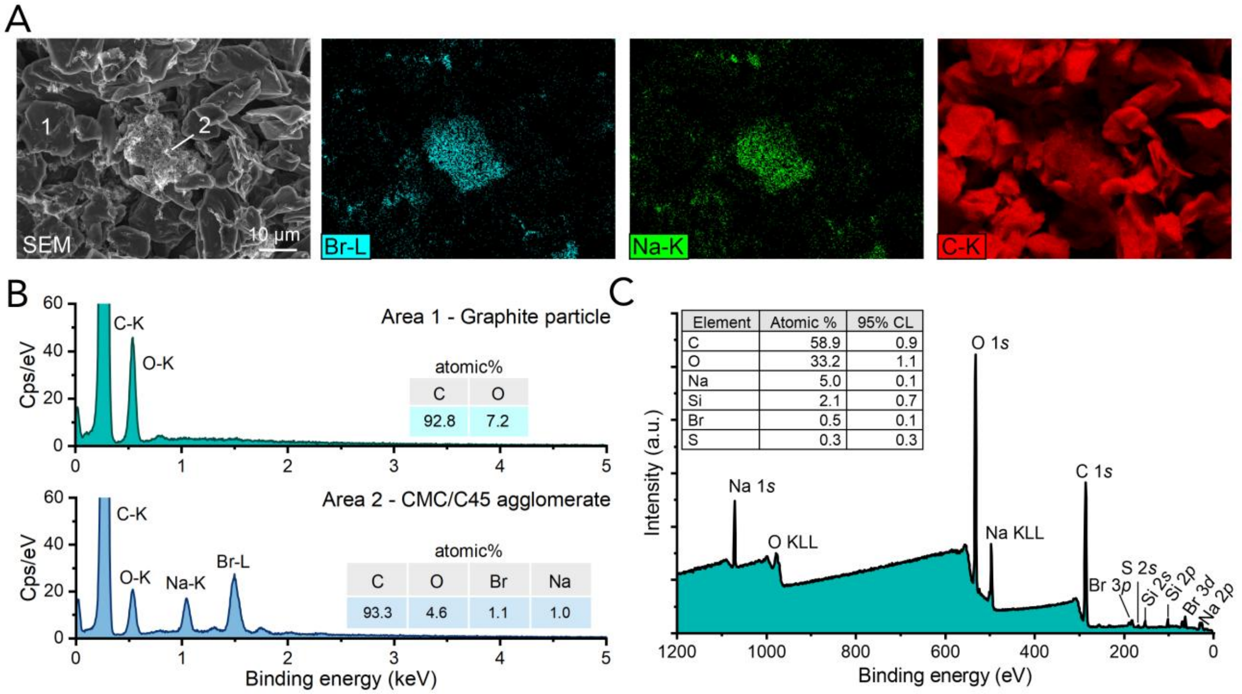

Figure S6. A) SEM image of a CMC/C45 agglomerate in the CMC-only graphitic electrode after bromination, together with EDX elemental maps of Br, Na and C. B) EDX quantitative analysis of the CMC/C45 agglomerate and a graphite particle (regions 1 and 2 in the A) SEM image, respectively). The 1:1 ratio of Br and Na within the CMC/C45 agglomerate suggests binding of Br to Na from Na-CMC and formation of NaBr with an increased quantity due to the extended surface area of CMC within the nanoporous agglomerate. The analysis was performed at a beam voltage of 10 kV. C) XPS survey spectrum and mean elemental composition of brominated CMC, based on measurements of n=3 spots of the same sample. The presence of Si may be due to the residuals of vacuum pump oil used during bromination, or from the cut glass slide used as a sample substrate.

Supplementary Note S5. Differences in drying-induced migration of SBR and CMC

To analyse differences in susceptibility of SBR and CMC to drying-induced migration, we first analysed top- and internal regions of Ag- and Br-stained single-binder electrodes containing either 1.5 wt.% CMC or 2.3 wt.% SBR as the only binder (the same electrodes as the ones described in Supplementary Note S3 before). The measurements of the top electrode surface were taken directly

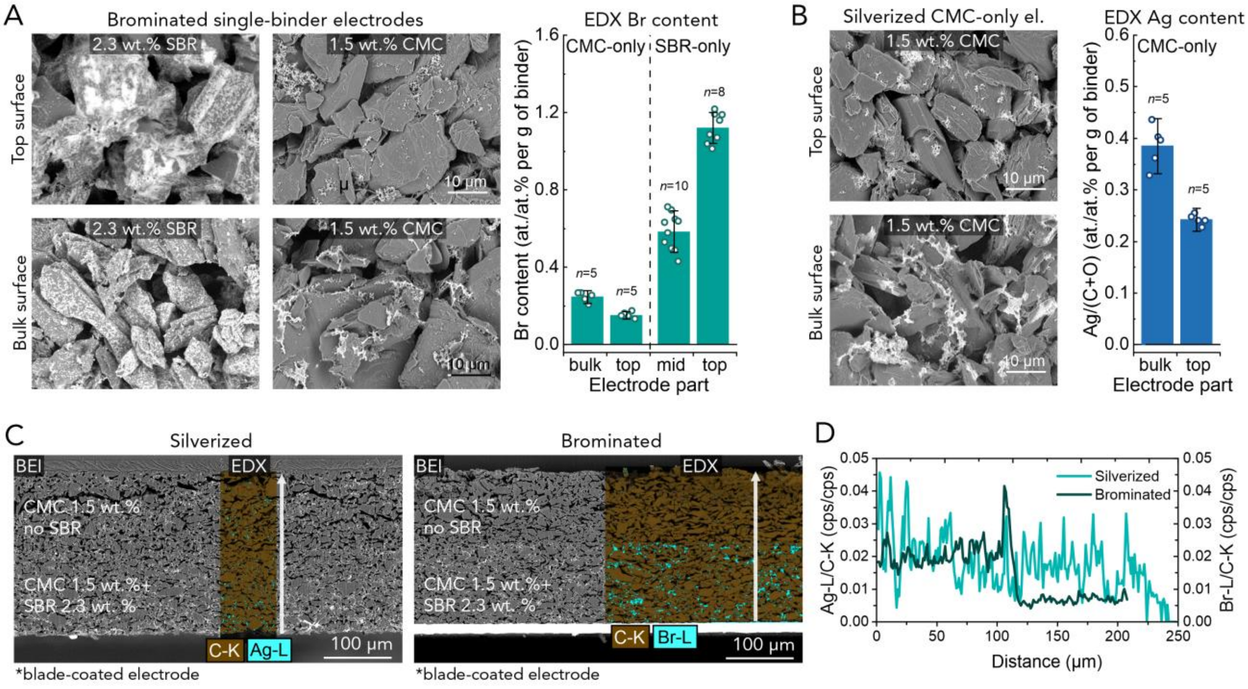

Figure S7. A) Backscattered electron imaging (BEI) of top and bulk surfaces of SBR- and CMC-only electrodes after bromination, together with the relative Br amount in these electrode regions quantified with EDX. B) BEI of top and bulk surfaces of CMC-only electrode after silverization, together with the relative Ag amount in these electrode regions quantified with EDX. In both A) and B), the Br and Ag EDX content was averaged from analyses of *n* random areas for each electrode part (the values of *n* are defined in the plots) and normalized to the average mass of binders within the electrodes. Error bars correspond to 95% confidence intervals of the average values. C) BEI images and EDX maps of silverized and brominated bi-layered electrodes designed to contain both CMC and SBR in the bottom layer and only CMC in the top layer. D) Through-thickness EDX profiles derived from the EDX maps in C). The electrode subject to silverization was transferred from the Cu foil substrate onto carbon adhesive tapes before Ag staining.

on the top surface of the electrodes after the staining, while the analysis of the bulk region of the electrodes was done by first removing the top electrode surface using sticky carbon SEM discs, followed by staining and EDX/BEI analysis of the exposed internal (bulk) region of the electrode.

Figure S7A shows BEI images of top- and internal parts of SBR- and CMC-only electrodes after bromination, together with Br content in these regions quantified with EDX. In the SBR-only electrode, a 2-times larger amount of Br was found on top of the electrode compared to the bulk of the electrode, suggesting a significant SBR concentration at the top electrode surface due to the drying-induced SBR migration. This is also evident in the BEI images showing large white

agglomerates of Br-SBR on top of the SBR-only electrode compared to somewhat less agglomerated Br-SBR inside this electrode. In the CMC-only electrode, on the other hand, the amount of Br was 1.6× lower in the top surface compared to the internal surface. This was identical to the 1.6× difference in the Ag content between the top and internal surfaces of the same type of electrode after silverization (Fig. S7B), confirming that the top surface of the CMC-only electrode contained 1.6× less CMC compared to the bulk of the electrode.

To further elucidate on the differences in CMC and SBR migration, using two-pass blade-coating we fabricated a bi-layered electrode in which the bottom-layer slurry contained both SBR and CMC, and the top-layer slurry contained only CMC. The cross-sectional BEI/EDX images of silverized and brominated electrodes after Ar-plasma polishing are shown in Fig. S7C, together with Ag and Br profiling in Fig. S7D. The images and elemental profiles in Fig. S7C and S7D show that Ag was roughly equally distributed in both layers, with a slightly decreasing Ag amount towards the top of the electrode. Although the Ag profile in Fig. S7D shows a significant noise due to the relatively small area of this sample analysed with EDX, the visibly mostly uniform BEI contrast in this electrode supports the Ag distribution derived with EDX. In the brominated sample, as expected, Br was detected mostly in the bottom layer (made from a SBR-containing slurry). A sharp peak with 2× larger Br amount is evident at the interface between the two layers in the Br profile in Fig S7D – a likely effect of SBR migration during the drying of the slurry-cast bottom layer. The BEI and EDX images of the brominated sample also show a small amount of bromine in the interparticle voids of the top layer that was deposited using a slurry without SBR. As described previously, the non-zero amount of Br in this layer was partially due to its surface binding with Na-CMC, but could also point to some amount of SBR migrating to the top electrode layer.

The results of staining these single-binder and bi-layered electrodes strongly suggest significant differences in migration tendency of SBR and CMC during electrode fabrication. The 2× larger Br amount on top of the SBR-only electrode compared to the depleted amount of SBR in the bulk of the electrode suggest that SBR in the SBR-only electorde was prone to a significant upward migration during electrode drying. This was also reflected by the SBR distribution in the bi-layered electrode, that showed doubling of Br amount at the interface between the SBR-containing lower layer and SBR-free layer, as well as non-zero Br amount in the upper layer. The CMC in the single-binder CMC-only electrode, on the other hand, seems to have retained a relatively large concentration within the bulk of the electrode, and even became somewhat depleted at the top electrode surface, which was also roughly reflected in the CMC distribution within the bi-layered sample. This could be related to the CMC concentration and retention in the smaller interparticle pores that typically experience delayed drying due to the large internal capillary forces.[31] Overall, the significantly stronger migration tendency of SBR compared to the CMC can result from the lower affinity of SBR to graphitic surfaces compared with CMC, aligning with the observations of Li and Lin who found preferential adsorption of CMC on graphite over SBR in wet slurries.[32] These results also support the closer-to-theoretical distribution of CMC compared with SBR in the bi-layer electrodes discussed in *Staining bi-layered electrodes* section of the main article, showing that the larger difference between the theoretical and measured SBR distribution in these electrodes was not due to lower accuracy of bromine staining, but due to the re-distribution of SBR during electrode manufacturing.

Supplementary Note S6. The effects of sample wetting during silverization

Because staining of CMC with Ag^+ is performed in an aqueous $AgNO_3$ solution, the wettability of the electrodes by the staining solution can influence the quantity of Ag binding to the CMC. In our work, we utilized two sample preparation and analysis routines, schematically presented in Figure S8A. In the first routine, the workflow consisted of first exposing sample's surface of interest (using e.g. scotch-tape delamination of the upper electrode surface), followed by silverization under mild vacuum (to facilitate surface wetting) and finally the analysis. This routine was applied for e.g. EsB binder surface imaging (described in the main article) or EDX/BEI silverization analysis of internal and top surfaces of non-homogenized samples described in Supplementary Note S3 and S5. The second routine consisted of first silverizing the sample under mild vacuum, followed by exposing the surface of interest (using e.g. Ar plasma cross-sectioning or homogenization by pulverization) and then the analysis. This routine was applied for through-thickness CMC analysis in electrodes (such as the bi-layered electrodes) and for CMC analysis in silverized and then homogenized samples described in the main article. Therefore, these two routines differed in the order of surface preparation and silverization – in the first routine, surface preparation was done before silverization, while in the second routine, silverization was performed before surface preparation.

The effects of wetting in the first staining routine were tested on electrodes containing 1.5 wt.% CMC that were first subject to surface preparation (e.g. scotch tape delamination to expose bulk sample surface), followed by silverization in 0.5M $AgNO_3$ with or without the addition of 1 mM Triton X-100 non-ionic surfactant. The addition of the surfactant singificantly lowers surface tension of the $AgNO_3$ solution, facilitating its penetration into the bulk of porous electrodes. Consequentially, if the analysis routine was sensitive to wetting, the experiments with and without

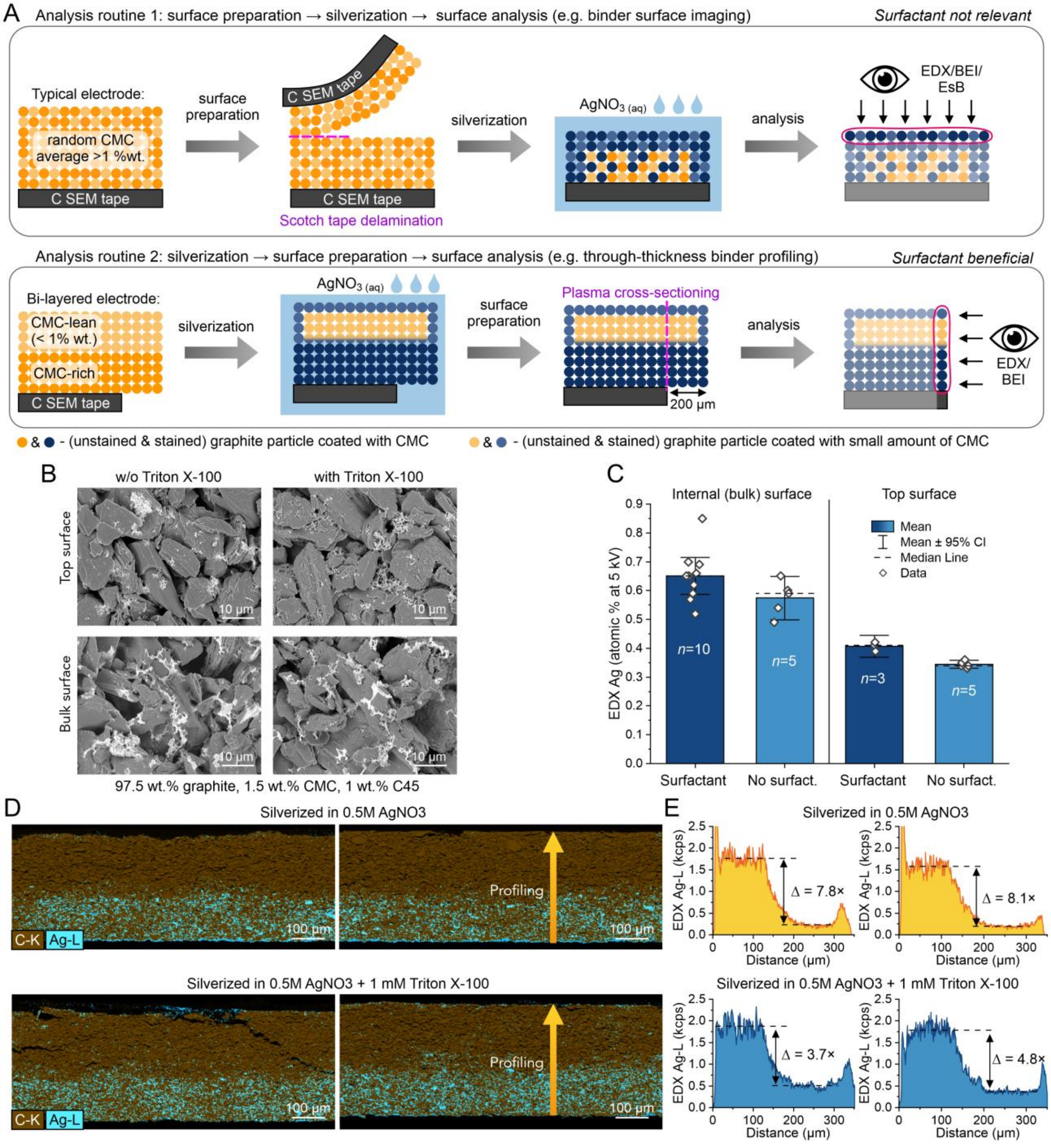

Figure S8. A) Schematics of sample preparation, silverization and analysis routine for through-thickness and top-down binder analysis. B) Top-down BEI images of top and internal (bulk) surfaces of an electrode silverized without or with the surfactant. The electrode contained 1.5 wt.% CMC and no SBR. C) EDX Ag quantification in internal- and top surfaces of the same type of electrode silverized without or with the surfactant. The number of technical replicates is indicated with *n* for each measurement. D) EDX maps of carbon (brown) and silver (teal) in bi-layered electrodes silverized without and with an addition of 1 mM Triton X-100 non-ionic surfactant to the 0.5M $AgNO_3$ staining bath. The electrodes contained four times more CMC and SBR in the lower layer compared with the upper layer. E) Ag-L profiles derived from the EDX maps, showing the ratio (*Δ*) of the Ag-L signal between the binder-rich and -lean layers.

the surfactant should yield singificantly different results. Fig. S8B shows BEI images acquired on the top and internal surfaces of these electrodes, showing that the addition of the surfactant did not affect the morphology of the silverized CMC. There was also only a minor increase of the Ag quantity detected on the surfaces silverized with the surfactant, compared to the ones silverized without it, as shown in the EDX Ag quantification in Fig. S8C. Therefore, the addition of the surfactant did not significantly influence Ag-CMC staining on such samples, which had their surface of interest prepared before the silverization, and where the mild vacuum applied during silverizaiton provided sufficient wetting of the sample surfaces directly exposed to the $AgNO_3$ solution. This is also shown in the scheme of the first analytical routine in Fig. S8A, where the unobstructed contact of the surface of interest with the staining solution leads to its efficient silverization. Note that in such case, even if some deeper regions of the sample are not wet by the staining solution, it does not affect the results of EDX/BEI analysis which is performed on the upper surface where the wetting and silverization is efficient.

The effects of wetting during the second staining routine were verified on bi-layered electrodes with a 4-times difference in binder fraction between a lower, binder-rich layer (with 4.5% SBR and 3.0% CMC by weight) and an upper, binder-lean layer (with 1.2% SBR and 0.8% CMC). To avoid galvanic replacement reactions between the $AgNO_3$ staining solution and metallic contaminants adventitiously deposited on the samples during plasma cross-section preparation, these samples had to be silverized first, followed by cross-section preparation – classifying them within the second analytical routine shown in Fig. S8A. Fig. S8D shows EDX maps of the samples silverized with and without the surfactant addition, with the derived through-thickness Ag profiles presented in Fig. S8E. The EDX maps and profiles show that in the CMC-rich region of the samples, silverization was similarly effective with and without the addition of the surfactant,

giving a similar amount of Ag-L signal in both cases. In the CMC-lean region of the samples, however, the samples silverized without the surfactant had a ~2× lower Ag-L intensity compared to the same region in the samples silverized with the surfactant. Consequently, the samples silverized without the surfactant had an amplified Ag-L ratio between the CMC-reach and -lean sample regions of ~8.0×, which is a 100% difference compared to the theoretical ratio of 4× and 3.5-4.8× in the samples silverized with the surfactant. This indicated an insufficient wetting and penetration of the CMC-lean (and thus, more hydrophobic) upper region of the samples by the pure $AgNO_3$ solution, which was effectively alleviated by the addition of the surfactant to the staining bath.

Such bi-layered samples present a combination of two conditions that made the CMC quantification strongly dependent on the wetting. First, these samples contained a large upper electrode region with a very low (<1% wt.) CMC content, making it inherently hydrophobic and difficult to wet. Second, the specific preparation of these samples relied on sufficient penetration of the staining solution to the deeper, bulk part of the samples, which could be exposed for the EDX analysis only after the staining (in this case, using plasma cross-sectioning). This is also visualized in the scheme of the second analytical routine in Fig. S8A, showing that in the samples with a very small CMC content and requiring silverization prior to the preparation of the analytical surface, insufficient inflitration of the staining solution in the CMC-lean sample regions may lower staining efficienty and the subsequent Ag amount detected by EDX or BEI in such regions. In these specific analytical cases, the addition of a surfactant to the staining solution can enhance bulk sample wetting beyond that provided by the mild vacuum applied during silverization, allowing for more accurate binder profiling, as also evident from Fig. S8D and S8E.

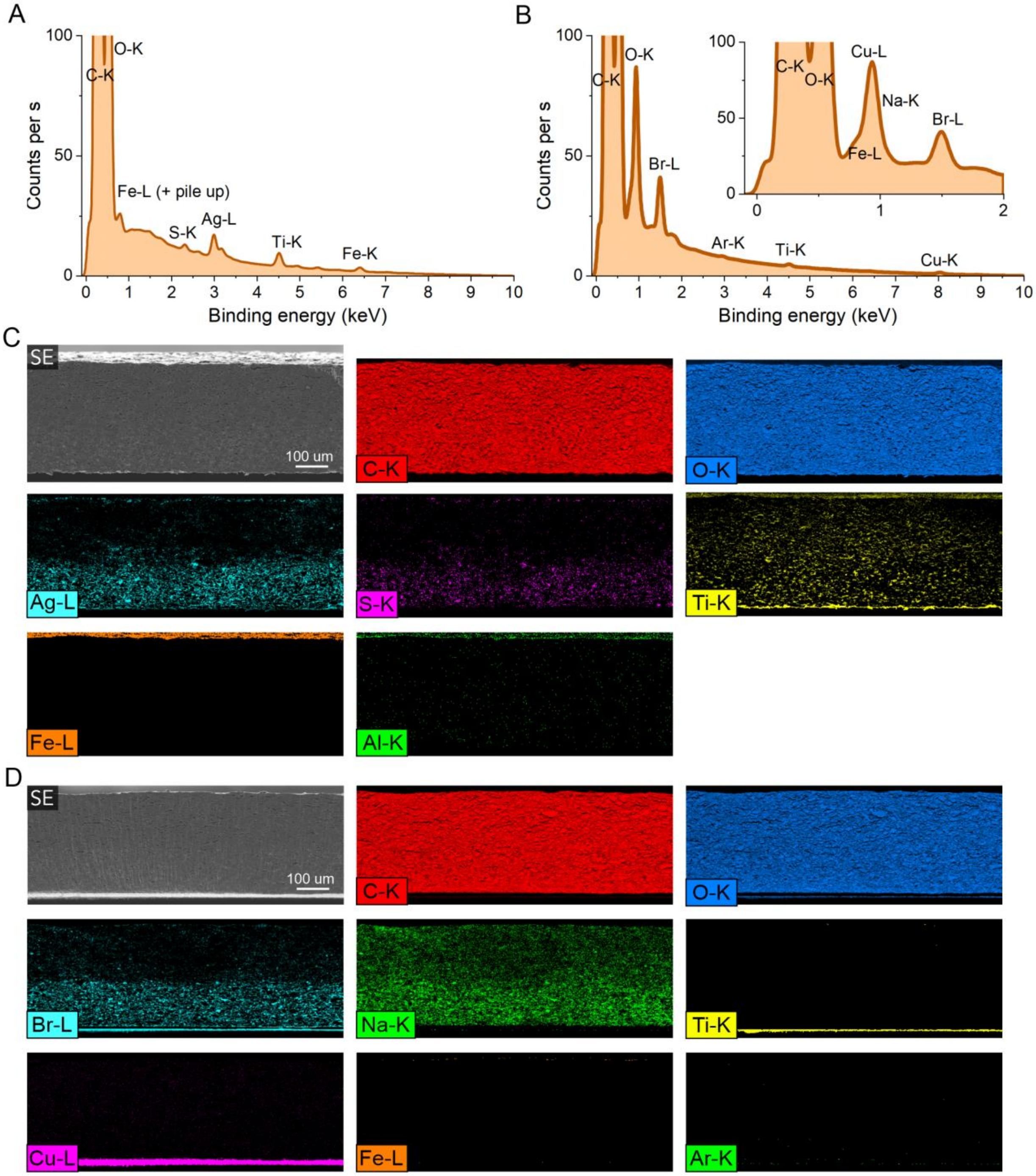

Figure S9. A) EDX spectrum of silverized bilayer electrode containing four times more CMC and SBR in the lower layer compared with the upper layer, after sample cross sectioning using ion polishing (the electrode is also shown in C). B) EDX spectrum of the same type of bilayer electrode after bromination and ion polishing (the electrode is also shown in D). C) Full EDX elemental mapping of silverized bi-layer electrode showing presence of metallic contaminants: Ti within and Fe on top of the ion-polished electrode. D) Full EDX elemental mapping of brominated bi-layer electrode.

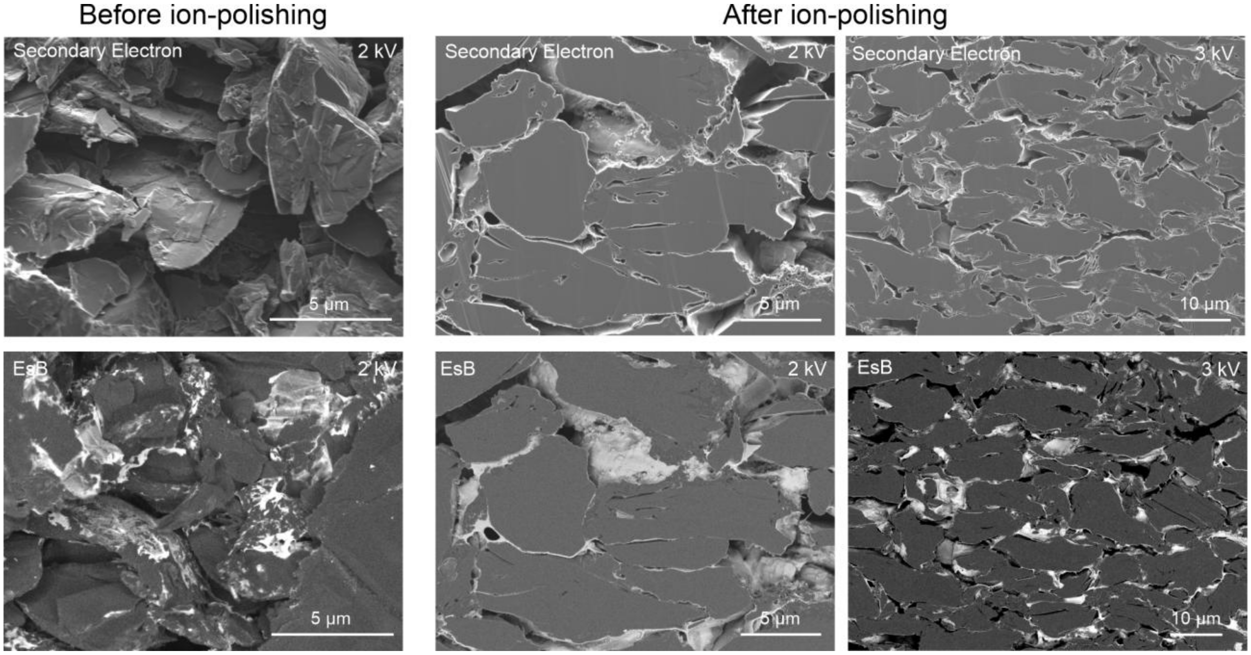

Figure S10. Cross-section secondary electron- and energy-selective backscattered electron images of silverized and calendered graphitic electrode before and after ion polishing. Ion polishing results in masking of the stained CMC (visible on particle surfaces before ion polishing) by the adventitious EsB contrast (seen on the interpore surfaces of graphitic particles after polishing). This is due to metallic contamination (mostly, Ti) present in the electrodes after ion polishing, detected with EDX analysis of the entire electrode after ion polishing – see also Fig. S7. The images were taken on the binder-rich fragments of bi-layer electrode used in the staining sensitivity study (containing 3% wt. CMC and 4.5% wt. SBR). Note that due to the increased content of CMC in the sample, the thicker CMC layer was detectable at 2 kV (left EsB image). The adventitious contrast was also visible in EsB images of every other ion-milled sample.

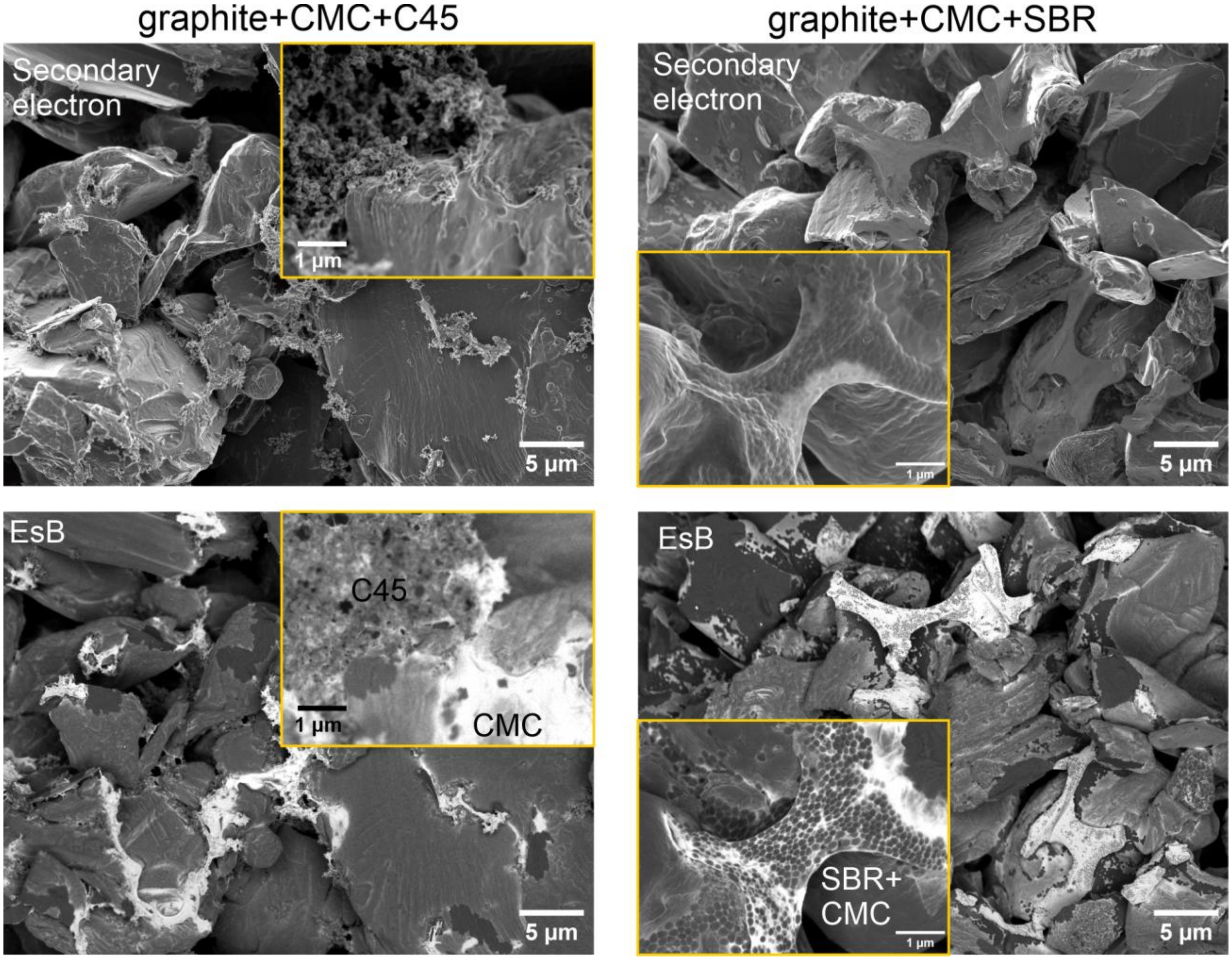

Figure S11. Secondary electron and EsB images of silverized graphitic electrodes containing CMC and C45 but no SBR (left column) or CMC and SBR but no C45 (right column). In the EsB images of SBR-free electrode on the bottom left, white CMC agglomerates do not contain the dark spheres that appear in the electrode made with SBR (on the bottom right), which allowed identification of these spheres as SBR nanoparticles. All images were acquired at 1 kV beam voltage and -513 V EsB grid voltage.

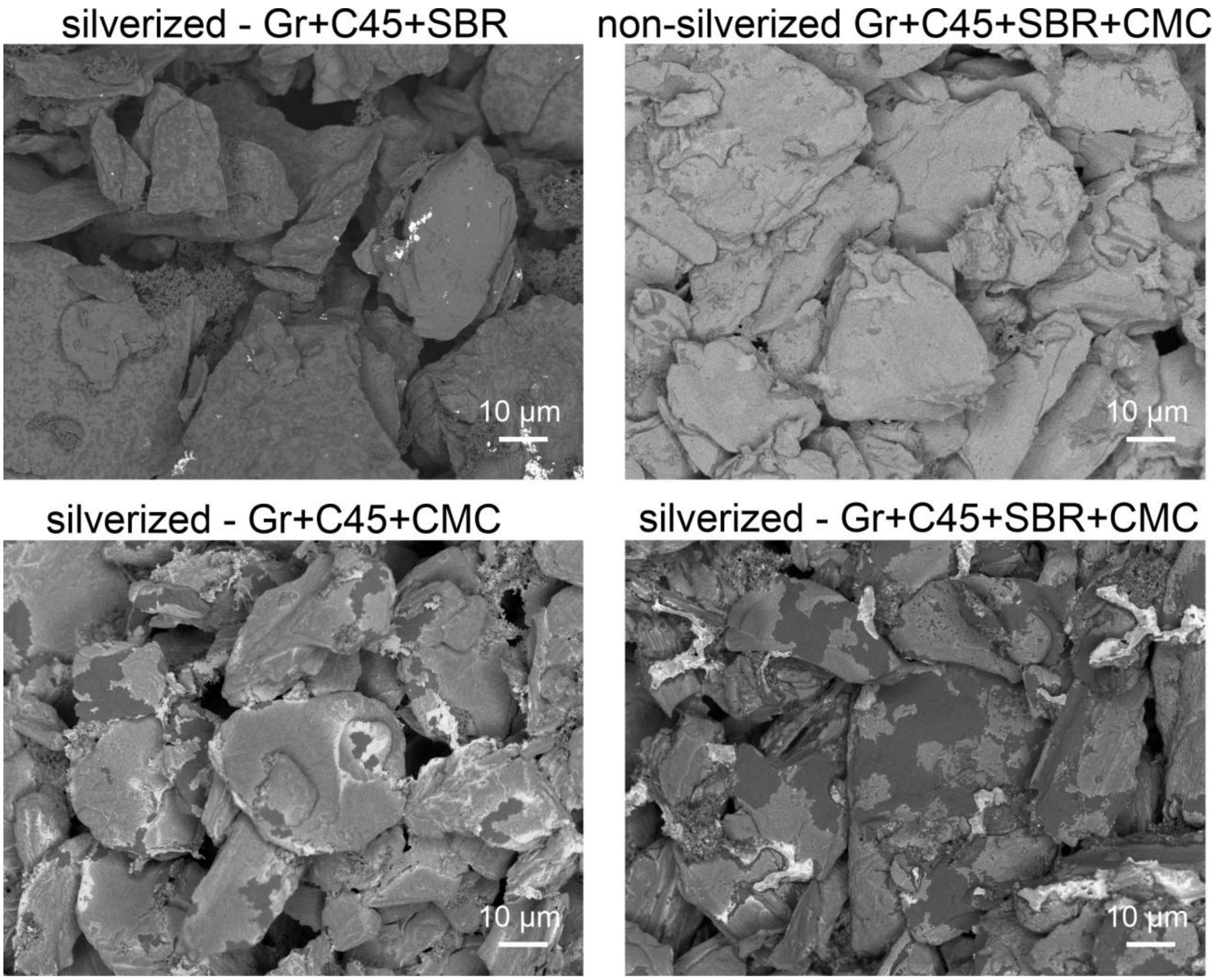

Figure S12. Comparative EsB images of silverized graphitic electrodes with and without CMC, as well as of a full graphitic electrode before silverization, showing the appearance of a white binder layer morphology only in the silverized electrodes containing CMC. All images were acquired at 1 kV beam voltage and -513 V EsB grid voltage and represent internal fragments of electrodes after delaminating their top parts using scotch tape. In the Gr+C45+SBR image, the few white areas were due to Ag^{+} binding to residual SBR polymerization by-products, as explained in Supplementary Note S2 and S3.

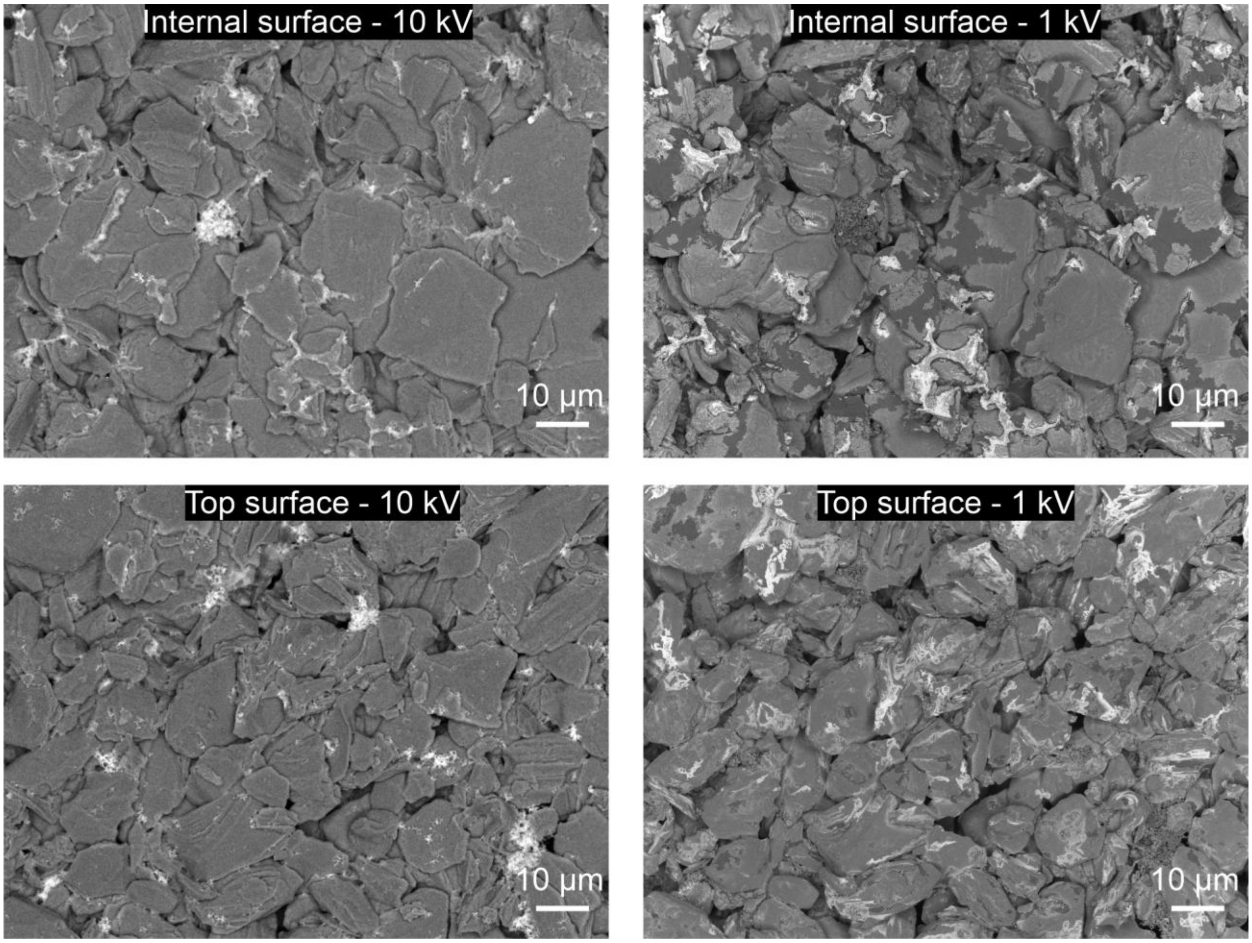

Figure S13. EsB images of the internal and top surface of graphitic electrodes after silverization, acquired at 10 kV and 1 kV of beam voltage and -513 V of EsB grid voltage

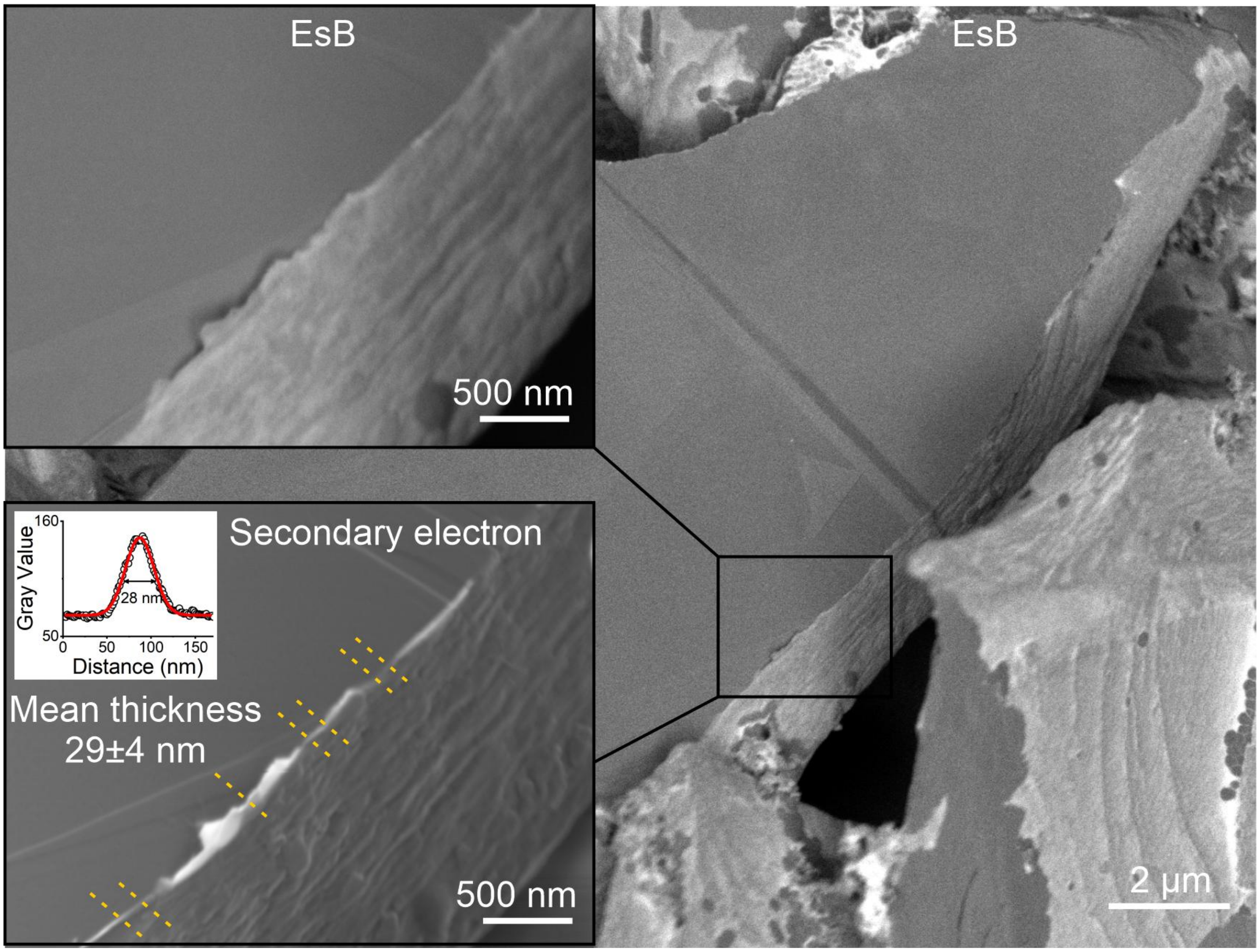

Figure S14. SEM and EsB images of a fractured graphitic particle inside a calendered and silverized electrode, showing dark internal particle surface next to the external particle surface coated by a white silverized CMC layer. The high resolution SEM image in the bottom inset was used for estimation of the CMC layer thickness. This was done by plotting pixel gray values along the paths indicated by the orange lines and extracting full width at half maximum of the fitted Gaussian peak. In total, n=9 measurements were performed from the same image. The error bar of the mean thickness corresponded to the 95% confidence interval. Note that thickness measurements could be affected by the tilt of the particle with respect to the SEM aperture, as well as curling of the CMC layer at the boundary of particle cross-section.

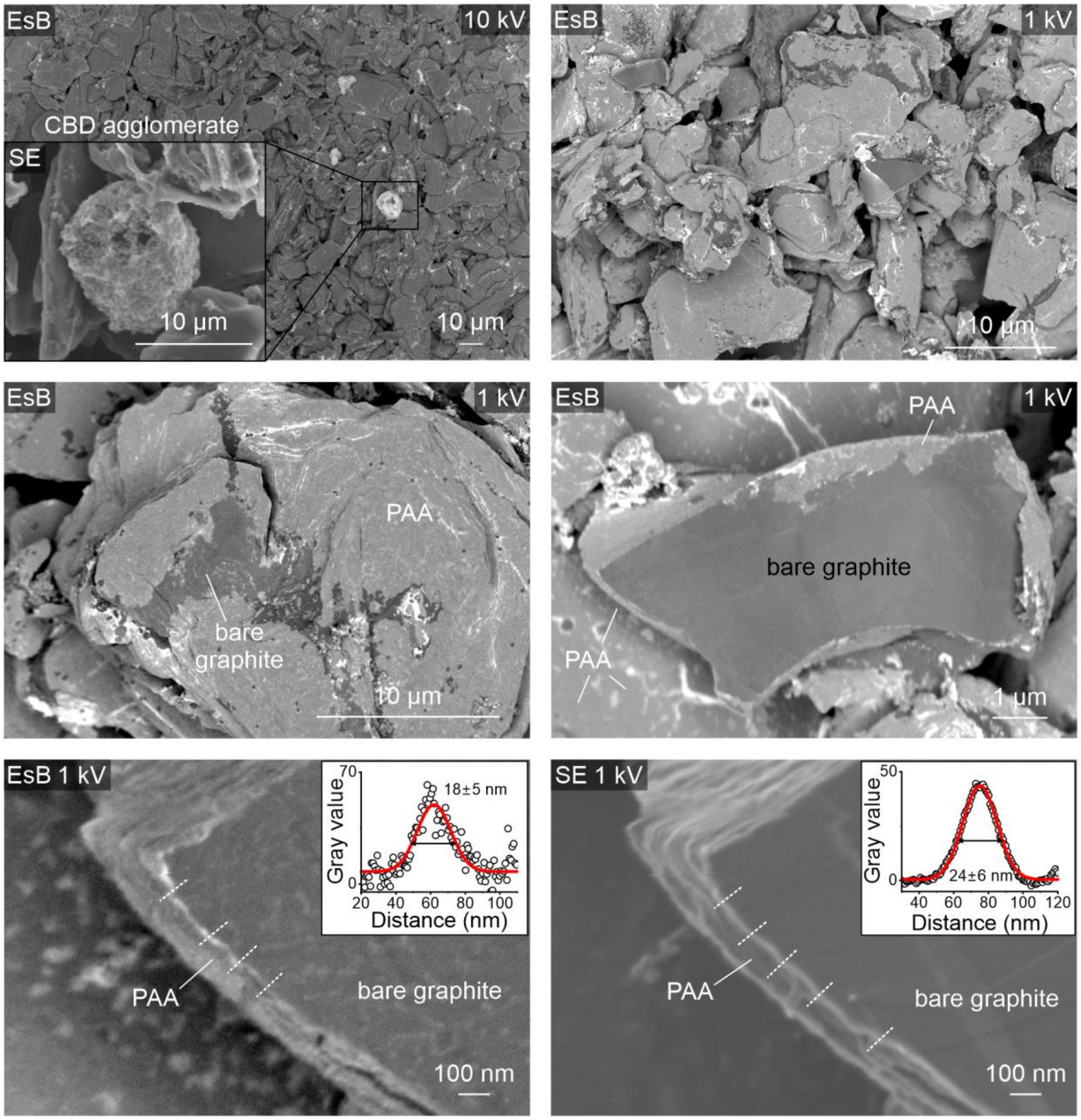

Figure S15. EsB and secondary electron (SE) imaging of internal surface of a silverized electrode consisting of 1.5 wt.% polyacrylic acid (PAA), 95.2 wt.% graphite, 2.3 wt.% SBR and 1 wt.% C45. The images were taken at 10 kV (top left) or 1 kV (all the other images) of primary electron beam voltage and show that PAA adapted the same type of morphologies on graphite as CMC. The high magnification images at the bottom show fractured graphite particle and estimated PAA layer thickness, based on *n*=4 (EsB) and 5 (SE) measurements along the dotted lines. The average PAA layer thickness is similar to the one of CMC in an analogous electrode (Figure S14).

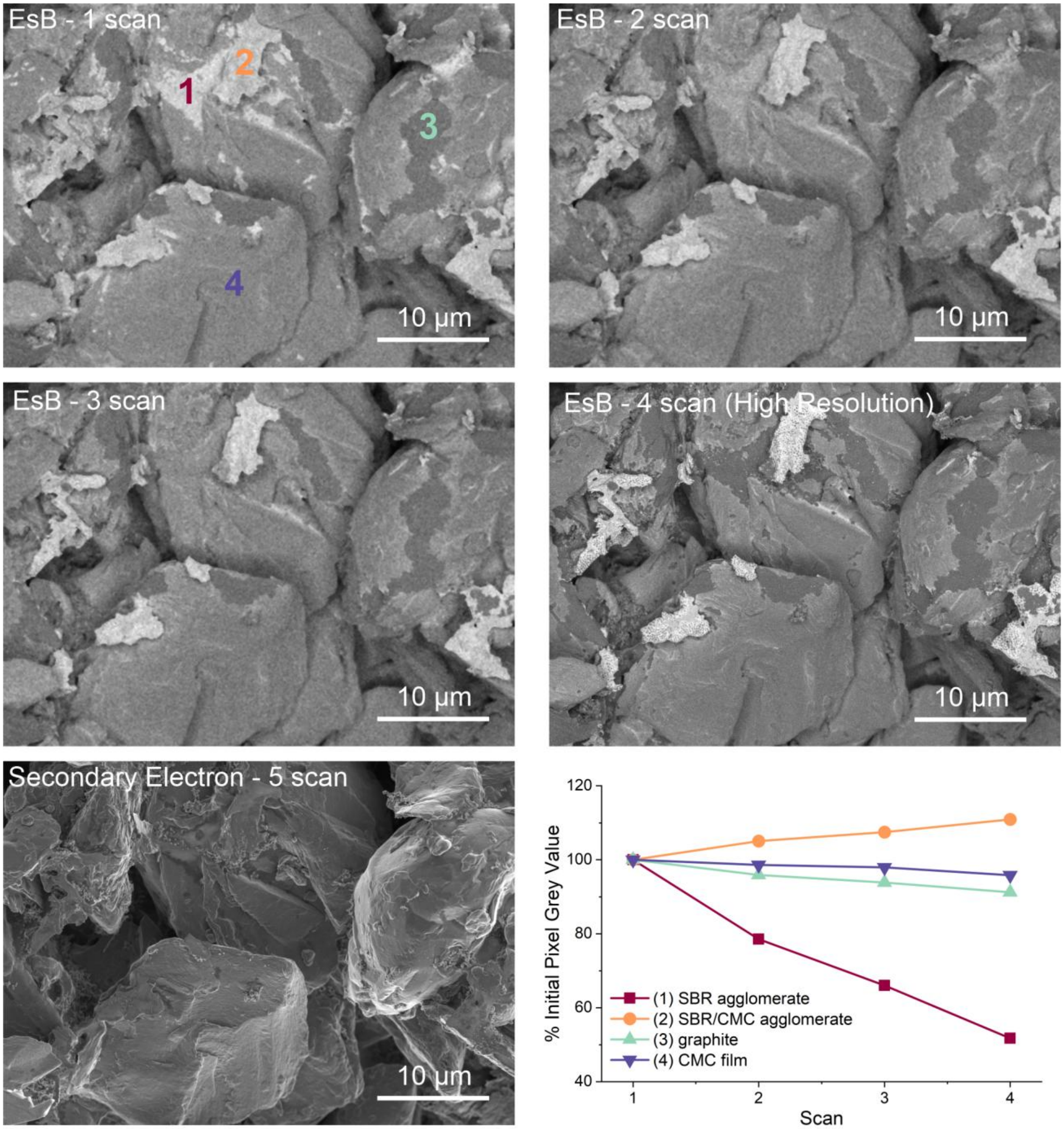

Figure S16. EsB and SEM images of internal electrode surface after bromination, acquired in consecutive imaging scans. The electrode had a standard composition of 95.2 wt% graphite, 2.3 wt.% SBR, 1.5 wt.% CMC, 1 wt.% C45. The bottom right graph shows a change in EsB contrast in consecutive images quantified in the four annotated areas corresponding to: (1) SBR particle agglomerate, (2), SBR/CMC agglomerate, (3) bare graphite surface and (4) graphite surface coated with continuous CMC film. All images were acquired at a beam voltage of 1 kV, beam current of 500 pA and EsB grid voltage of -513 V. The first 3 EsB images were acquired using dwell time of 13 ms/pixel and the 4th EsB image with dwell time of 27 ms/pixel.

Calendered?	Coating	Samples	Thickness (µm)	Loading (mg/cm²)	Porosity (%)	Sheet resistance (Ω/sq.)	Resistivity (mΩ×cm)
No	Standard	5	130±5	13.4±0.6	53±1	9.7±0.3	127±2
No	Modified	5	121±9	12.8±0.8	51±1	9.0±0.6	109±3
Yes	Standard	8	228±5	33.7±0.7	32±1	6.1±0.4	141±8
Yes	Modified	8	227±4	33.1±0.5	33±1	5.1±0.6	114±14

Table S1. Properties of electrode coatings characterized with four-point probe measurement in *Correlating slurry mixing…* section of the article. The calendered coatings were fabricated separately to the uncalendared coatings.

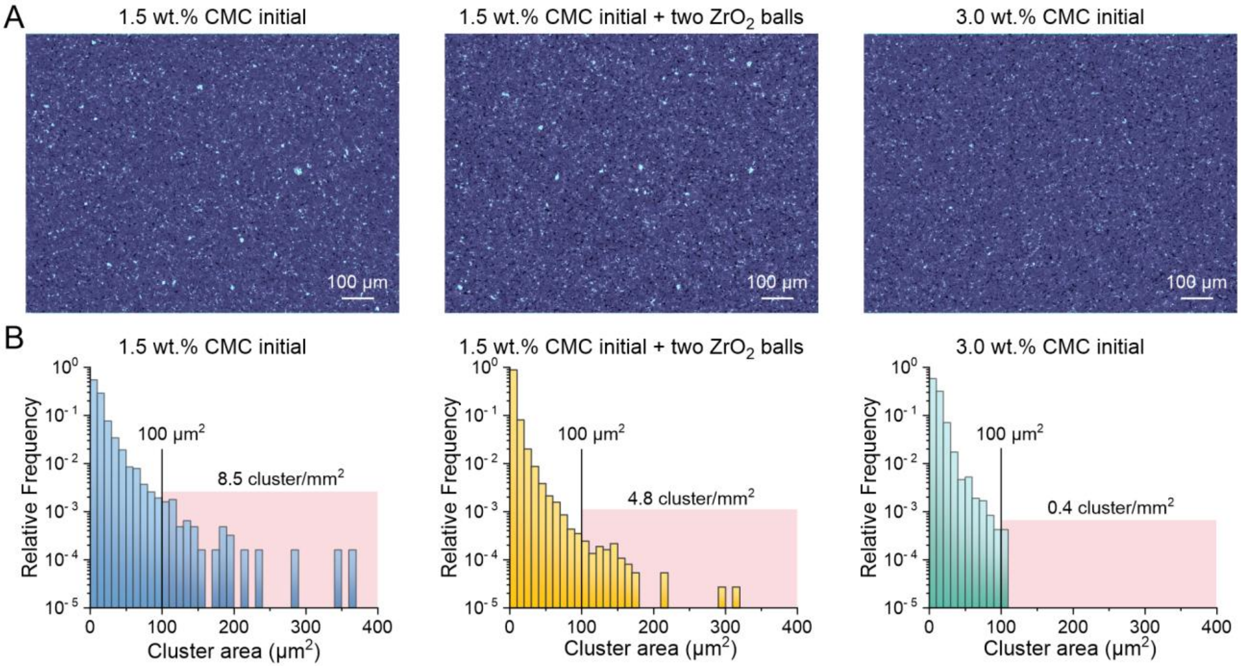

Figure S17. Comparison of carbon-binder domain (CBD) agglomeration in electrodes made from standard (1.5 wt. % CMC initial) slurry, the same type of slurry with an addition of two zirconia balls to the mixing pot, or the modified (3.0 wt.% CMC initial) slurry. (A) Representative color-enhanced backscattered electron images (BEI) of the top electrode surfaces, showing CBD as the bright blue phase; (B) Histograms showing size distribution of the CBD clusters in the three types of electrodes. BEI image segmentation and CBD clustering analysis was done according to Supplementary Procedure S2.

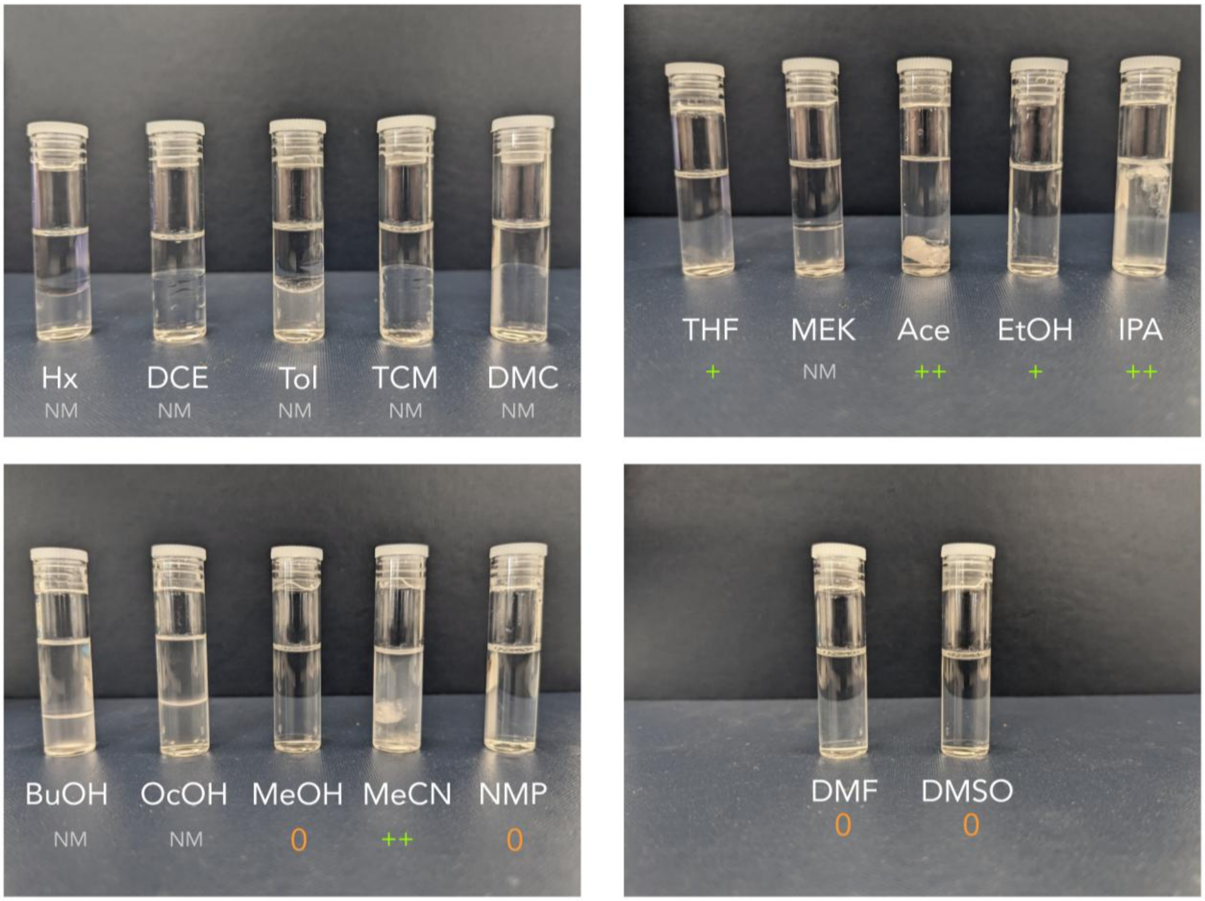

Figure S18. Screening of organic solvents for phase inversion of CMC from aqueous solution. The photographs show vials containing 1 mL of 1.5% aqueous carboxymethyl cellulose and approximately 2 mL of organic solvent after thorough mixing and equilibration over 3 nights. The solvent abbreviations are: Hx – hexane, DCE – trans-1,2-dichloroethylene, Tol – toluene, TCM – chloroform, DMC – dimethyl carbonate, THF – tetrahydrofuran, MEK – methyl ethyl ketone, Ace – acetone, EtOH – ethyl alcohol, IPA – isopropyl alcohol, BuOH – 1-butanol, OcOH – 1-octanol, MeOH – methanol, MeCN – acetonitrile, NMP – N-methyl-2-pyrrolidone, DMF – N,N-dimethylformamide, DMSO – dimethyl sulfoxide. The subscripts below each vial indicate the observations: NM – solvent not miscible with the aqueous CMC solution, 0 – solvent miscible but no precipitation of CMC, + - some precipitation of CMC, ++ - complete precipitation of CMC

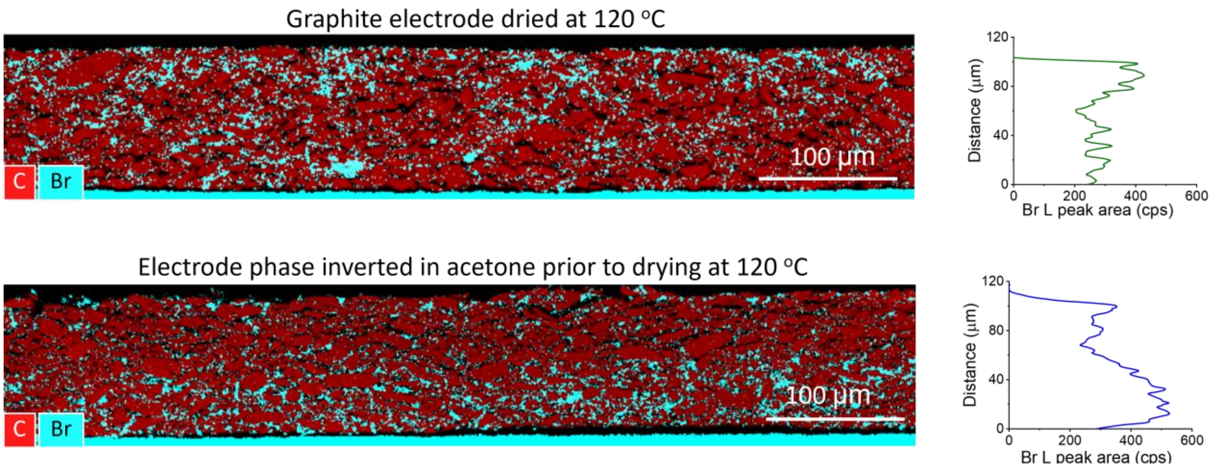

Figure S19. EDX maps of Br and C in reference and phase inverted in acetone electrodes after cross-sectioning and Br staining, together with through-thickness Br-L distribution profiles derived from the EDX maps. The profiles exclude the brominated Cu current collector region. The electrodes were separately made and identically processed as the electrodes described in the phase inversion section of the main article.

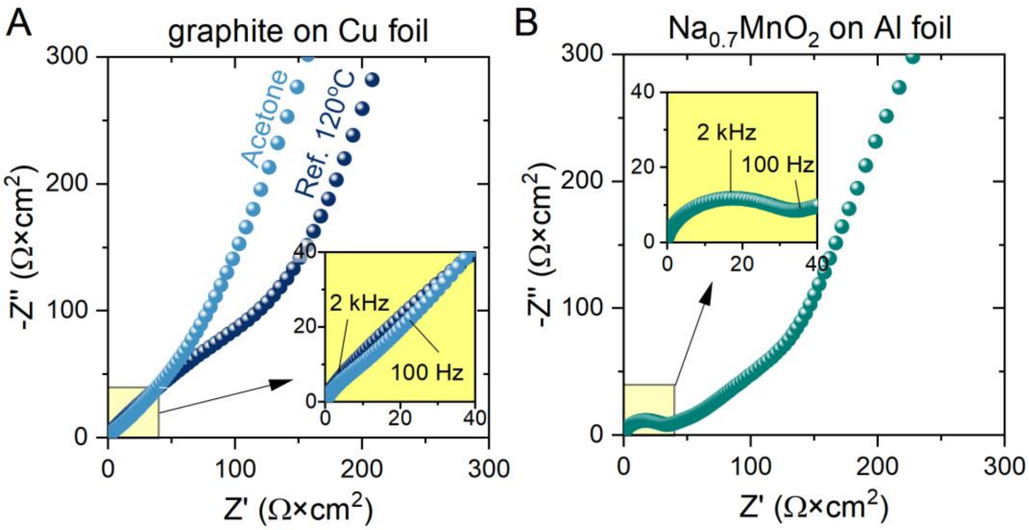

Figure S20. Nyquist plots comparing impedance response of: (A) calendered reference- and acetone-phase-inverted graphite/Cu electrodes described in this work and (B) calendered $Na_{0.7}MnO_2$/Al electrodes measured separately in our group. The insets show high frequency, low impedance regions of the spectra. The measurements were performed at open circuit potential, in symmetric cell configuration, in 10 mM tetrabutylammonium perchlorate in 1:1 wt. EC:DMC, in 100kHz – 10 mHz frequency range. The high-frequency resistive/capacitive semicircle in the $Na_{0.7}MnO_2$/Al electrode represents the contact resistance between the electrode coating and Al current collector that is characteristic to battery electrodes supported on metallic foils with a native oxide layer.[41,42] The lack of semicircles in the Nyquist plots of the graphite/Cu electrodes shows that the contact resistance was below detection limit in both the reference- and acetone-phase-inverted graphite electrodes. The plots were corrected for series resistance by shifting their origin to $Z' = 0\ \Omega\times cm^2$

Supplementary Note S7. Fiji (ImageJ) macro for quantifying SBR coverage from EsB images of Br-stained electrodes.

The macro quantifies SBR surface coverage from an initial and nth EsB image of an electrode fragment. Before running the macro, the initial and nth image is renamed as “1” and “2”, respectively. The macro requires installation of the EM Tool plugin made by Zhou Xu, available at https://imagej.net/plugins/imbalence.

```
//set global scale of images using EM Tool
selectImage("1");
run("SEM Zeiss metadata Scale");
selectImage("2");
run("SEM Zeiss metadata Scale");
getPixelSize(unit, pixelWidth, pixelHeight);
scale = 1/pixelWidth
run("Set Scale...", "distance=" + scale + " known=1 unit=nm global");

//Stack Images and Register
run("Images to Stack");
run("Linear Stack Alignment with SIFT", "initial_gaussian_blur=1.60 steps_per_scale_octave=3
minimum_image_size=64 maximum_image_size=1024 feature_descriptor_size=4
feature_descriptor_orientation_bins=8 closest/next_closest_ratio=0.92 maximal_alignment_error=5
inlier_ratio=0.05 expected_transformation=Affine interpolate");

//Crop images to remove registration artifacts at the edges
run("Duplicate...", "duplicate");
makeRectangle(5, 4, 1013, 758);
run("Crop");
run("Median...", "radius=1 stack");
run("Stack to Images");
selectImage("1");
run("Duplicate...", " ");
selectImage("2");
run("Duplicate...", " ");

//Apply Non-local Means Denoising to smooth images for thresholding
selectImage("1-1");
run("Non-local Means Denoising", "sigma=5 smoothing_factor=1");
//Duplicate 1st image to be used as a mask for removing holes
run("Duplicate...", " ");

selectImage("2-1");
run("Non-local Means Denoising", "sigma=5 smoothing_factor=1");

selectImage("1-2");
run("Auto Local Threshold", "method=Phansalkar radius=100 parameter_1=0.7 parameter_2=0 white");
run("Analyze Particles...", "  show=[Overlay] display summarize overlay");

//update standard deviations to the summary table
```

```
selectWindow("Results");
aa = Table.getColumn("Area");
run("Clear Results");
//close("Results");
Array.getStatistics(aa, mi, mx, mn, std);
selectWindow("Summary");
Table.set("STD Area", nResults, std);
Table.update;

//Duplicate 2nd image (outgassed) to be used as a mask for removing SBR/CMC agglomerates
selectImage("2-1");
run("Duplicate...", " ");

//Threshold 2nd outgassed image and extract area of large SBR/CMC agglomerates
setAutoThreshold("Yen dark");
//setThreshold(152, 255);
setOption("BlackBackground", true);
run("Convert to Mask");
run("Analyze Particles...", "size=125663.71-Infinity show=Masks display summarize overlay");

//update standard deviations to the summary table
selectWindow("Results");
aa = Table.getColumn("Area");
run("Clear Results");
close("Results");
Array.getStatistics(aa, mi, mx, mn, std2);
selectWindow("Summary");
Table.set("STD Area", nResults+1, std2);
Table.update;
selectImage("2-2");
run("Create Selection");

//Subtract large SBR/CMC agglomerates from images 1 and 2
selectImage("1-1");
run("Restore Selection");
//run("Make Inverse");
run("Clear", "slice");
run("Select None");
selectImage("2-1");
run("Restore Selection");
//run("Make Inverse");
run("Clear", "slice");
run("Select None");

//Subtract image 2 from 1
imageCalculator("Subtract create", "1-1","2-1");
run("Brightness/Contrast...");
run("Enhance Contrast", "saturated=0.35");
run("Apply LUT");
setAutoThreshold("Otsu dark");
//setThreshold(78, 255);
setOption("BlackBackground", true);
run("Convert to Mask");

selectImage("1-2");
setAutoThreshold("Yen");
```

```
    run("Create Selection");
    selectImage("Result of 1-1");
    run("Restore Selection");
    //run("Make Inverse");
    //setBackgroundColor(0, 0, 0);
    run("Clear", "slice");
    run("Select None");

    run("Analyze Particles...", "size=22698-Infinity show=[Overlay Masks] display summarize overlay");
    //update standard deviations to the summary table
    selectWindow("Results");
    aa = Table.getColumn("Area");
    run("Clear Results");
    close("Results");
    Array.getStatistics(aa, mi, mx, mn, std3);
    selectWindow("Summary");
    Table.set("STD Area", nResults+2, std3);
Table.update;
```

Supplementary Note S8. Jupyter Notebook Python code for extracting backscattered electron trajectories from the output files of Casino 3.3 Monte Carlo simulations.

The code extracts the simulated trajectories of backscattered electrons from the .dat files that can be exported from Casino 3.3 after performing the simulations. For the code to work correctly, the files need to be saved with the following options checked in the Casino export window:

a) Scan Point Options: *X* and *Y* checked, rest unchecked
b) Collision Options: *X*, *Y*, *Z*, Energy, Collision Type, Region ID checked, rest unchecked
c) Collision Type Options: Collisions with atoms, Collisions with Regions checked, rest unchecked

All the remaining options in the export window need to be unchecked.

```
# The paths to the the input and output files are defined at the end of the code

import pandas as pd
def parse_data(file_path):
    with open(file_path, 'r') as file:
        lines = file.readlines()

    lines = lines[10:]  # Skip the first 10 lines
```

```
    scan_points = []
    current_scan_point = {}
    current_trajectory = {}
    collisions_header = ["X", "Y", "Z", "Energy", "CollisionType", "Region_ID"]
    collisions_data = []
    reading_collisions = False

    for line in lines:
        stripped_line = line.strip()

        if "Scan Point" in stripped_line and "Trajectories :" not in stripped_line:
            if current_scan_point:
                if current_trajectory:
                    current_trajectory["Collisions"] = collisions_data
                    current_scan_point["Trajectories"].append(current_trajectory)
                scan_points.append(current_scan_point)
                collisions_data = []
                current_trajectory = {}
            sp_number = stripped_line.split('\t')[1]
            current_scan_point = {"Scan Point": sp_number, "Trajectories": [], "X": None, "Y": None}

        elif '"X"' in stripped_line and not current_trajectory:  # X coordinate for scan point
            current_scan_point["X"] = stripped_line.split('\t')[1]

        elif '"Y"' in stripped_line and not current_trajectory:  # Y coordinate for scan point
            current_scan_point["Y"] = stripped_line.split('\t')[1]

        elif "Trajectory" in stripped_line:
            if current_trajectory:
                current_trajectory["Collisions"] = collisions_data
                current_scan_point["Trajectories"].append(current_trajectory)
                collisions_data = []
            traj_number = stripped_line.split('\t')[1]
            current_trajectory = {"Trajectory": traj_number, "Collisions": []}

        elif "Collisions" in stripped_line:
            reading_collisions = True

        elif reading_collisions and stripped_line and stripped_line.split()[0].replace('.', '', 1).isdigit():
            collision_data = stripped_line.split()
            if len(collision_data) == len(collisions_header):  # Ensuring proper data row
                collisions_data.append(dict(zip(collisions_header, collision_data)))

    # Finalizing the last entries
    if collisions_data:
        current_trajectory["Collisions"] = collisions_data
    if current_trajectory:
        current_scan_point["Trajectories"].append(current_trajectory)
    if current_scan_point:
        scan_points.append(current_scan_point)

    return scan_points

def create_collision_table_with_coords(parsed_data):
    columns = ["Scan Point number", "Scan Point X", "Scan Point Y", "Trajectory number", "X", "Y", "Z", "Energy"]
    rows = []
```

```
    for scan_point in parsed_data:
        sp_number = scan_point["Scan Point"]
        sp_x = scan_point["X"]
        sp_y = scan_point["Y"]

        for trajectory in scan_point["Trajectories"]:
            traj_number = trajectory["Trajectory"]

            for collision in trajectory["Collisions"]:
                row = [
                    sp_number,
                    sp_x,
                    sp_y,
                    traj_number,
                    collision["X"],
                    collision["Y"],
                    collision["Z"],
                    collision["Energy"]
                ]
                rows.append(row)

    return pd.DataFrame(rows, columns=columns)

def process_file(file_path, output_path):
    parsed_data = parse_data(file_path)
    df = create_collision_table_with_coords(parsed_data)
    df.to_csv(output_path, index=False)

# File paths
input_file = # Path to the .dat file output from the Casino simulations, including the name of the input file.dat
output_file = # Path where the output .csv file is to be stored, including the name of the output file.csv

# Process the file
process_file(input_file, output_file)
```

Supplementary References

1. International Organization for Standardization (2016), Styrene-butadiene rubber (SBR) — Determination of the microstructure of solution-polymerized SBR — Part 2 : FTIR with ATR method (ISO Standrad No. 21561-2:2016). https://www.iso.org/standard/64125.html (2016).
2. Guilment, J. & Bokobza, L. Determination of polybutadiene microstructures and styrene-butadiene copolymers composition by vibrational techniques combined with chemometric treatment. *Vib. Spectrosc.* **26**, 133–149 (2001).
3. Munteanu, S. B. & Vasile, C. Spectral and thermal characterization of styrene-butadiene copolymers with different architectures. *J. Optoelectron. Adv. Mater.* **7**, 3135–3148 (2005).
4. Ul-Hamid, A., Soufi, K. Y., Al-Hadhrami, L. M. & Shemsi, A. M. Failure investigation of

an underground low voltage XLPE insulated cable. *Anti-Corrosion Methods Mater.* **62**, 281–287 (2015).

5. Cuba-Chiem, L. T., Huynh, L., Ralston, J. & Beattie, D. A. In situ particle film ATR FTIR spectroscopy of carboxymethyl cellulose adsorption on talc: Binding mechanism, pH effects, and adsorption kinetics. *Langmuir* **24**, 8036–8044 (2008).

6. Zhang, F., Dou, J. & Zhang, H. Mixed membranes comprising carboxymethyl cellulose (as capping agent and gas barrier matrix) and nanoporous ZIF-L nanosheets for gas separation applications. *Polymers (Basel).* **10**, (2018).

7. Gorgieva, S. & Kokol, V. Synthesis and application of new temperature-responsive hydrogels based on carboxymethyl and hydroxyethyl cellulose derivatives for the functional finishing of cotton knitwear. *Carbohydr. Polym.* **85**, 664–673 (2011).

8. Nuisin, R., Siripongpreda, T., Watcharamul, S., Siralertmukul, K. & Kiatkamjornwong, S. Facile Syntheses of Physically Crosslinked Carboxymethyl Cellulose Hydrogels and Nanocomposite Hydrogels for Enhancing Water Absorbency and Adsorption of Sappan Wood Dye. *ChemistrySelect* **7**, (2022).

9. Papageorgiou, S. K. *et al.* Metal-carboxylate interactions in metal-alginate complexes studied with FTIR spectroscopy. *Carbohydr. Res.* **345**, 469–473 (2010).

10. Kabachkov, E. N., Baskakov, S. A. & Shulga, Y. M. Degradation of Polymer Films of Sodium Alginate during Prolonged Irradiation with X-ray under Ultra-High Vacuum. *Polymers (Basel).* **16**, 1–14 (2024).

11. Vasil'kov, A. *et al.* Cellulose-based hydrogels and aerogels embedded with silver nanoparticles: Preparation and characterization. *Gels* **7**, (2021).

12. Miyama, T. & Yonezawa, Y. Photoinduced formation and aggregation of silver nanoparticles at the surface of carboxymethylcellulose films. *J. Nanoparticle Res.* **6**, 457–465 (2004).

13. Beamson, G. & Briggs, D. *High Resolution XPS of Organic Polymers. The Scienta ESCA300 Database*. (Wiley, 1992). doi:10.1002/adma.19930051035.

14. Jeschull, F. *et al.* Interphase formation with carboxylic acids as slurry additives for Si electrodes in Li-ion batteries. Part 2: a photoelectron spectroscopy study. *J. Phys. Energy* **5**, 025002 (2023).

15. Bañuls-Ciscar, J., Abel, M.-L. & Watts, J. F. Characterisation of cellulose and hardwood organosolv lignin reference materials by XPS. *Surf. Sci. Spectra* **23**, 1–8 (2016).

16. Ramstedt, M. & Shchukarev, A. XPS spectra from five monosaccharides. *Surf. Sci. Spectra* **31**, (2024).

17. Gerin, P. A., Genet, M. J. & Rouxhet, P. G. Polysaccharide by XPS: Analysis of Maltodextrin. *Surf. Sci. Spectra* **4**, 28–32 (1996).

18. Martínez-Rodríguez, M. A. *et al.* Influence of polysaccharides' molecular structure on the antibacterial activity and cytotoxicity of green synthesized composites based on silver nanoparticles and carboxymethyl-cellulose. *Nanomaterials* **10**, 1–18 (2020).

19. Pinder, J. W. *et al.* Avoiding common errors in X-ray photoelectron spectroscopy data collection and analysis, and properly reporting instrument parameters. *Appl. Surf. Sci. Adv.* **19**, (2024).

20. Greczynski, G. & Hultman, L. Compromising Science by Ignorant Instrument Calibration—Need to Revisit Half a Century of Published XPS Data. *Angew. Chemie - Int. Ed.* **59**, 5002–5006 (2020).

21. Ferraria, A. M., Carapeto, A. P. & Botelho Do Rego, A. M. X-ray photoelectron spectroscopy: Silver salts revisited. *Vacuum* **86**, 1988–1991 (2012).

22. Zhang, Y. *et al.* Ag clusters electrochemically reduced in stearic acid Langmuir-Blodgett films and their structural characterizations. *Sci. China, Ser. B Chem.* **40**, 397–404 (1997).

23. Moulder, J. F., Stickle, W. F., Sobol, P. E. & Bomben, K. D. *Handbook of X-ray Photoelectron Spectroscopy*. (Physical Electronics Inc., 1995).

24. Gelius, U. *et al.* Molecular Spectroscopy by Means of ESCA III. Carbon Compounds. *Phys. Scr.* **2**, 70–80 (1970).

25. Chanunpanich, N. *et al.* Surface modification of polyethylene through bromination. *Langmuir* **15**, 2089–2094 (1999).

26. *Next Generation Electrodes - Nextrode*. https://nextrode.web.ox.ac.uk/ (2025).

27. Quirk, R. P. & Pickel, D. L. Polymerization: Elastomer Synthesis. in *The Science and Technology of Rubber* (eds. Mark, J. E., Erman, B. & Roland, M. C.) 47–51 (Elsevier, 2005). doi:10.1016/B978-012464786-2/50005-5.

28. Terlingen, J. G. A., Feijen, J. & Hoffman, A. S. Immobilization of Surface Active Compounds on Polymer Supports Using Glow Discharge Processes. *J. Colloid Interface Sci.* **155**, 55–65 (1993).

29. Sinha, T., Gude, V. & Rao, N. V. S. Synthesis of Silver Nanoparticles Using Sodium Dodecylsulphate. *Adv. Sci. Eng. Med.* **4**, 381-387(7) (2012).

30. Varenikov, A., Shapiro, E. & Gandelman, M. Decarboxylative Halogenation of Organic Compounds. *Chem. Rev.* **121**, 412–484 (2021).

31. Altvater, A. *et al.* Application of Multistage Drying Profiles for Accelerated Production of Li-Ion Battery Anodes Using Infrared Radiation: Validation with Electrochemical Performance and Structural Properties. *Energy Technol.* **12**, (2024).

32. Li, C. C. & Lin, Y. S. Interactions between organic additives and active powders in water-based lithium iron phosphate electrode slurries. *J. Power Sources* **220**, 413–421 (2012).

33. Fairley, N. *et al.* Systematic and collaborative approach to problem solving using X-ray photoelectron spectroscopy. *Appl. Surf. Sci. Adv.* **5**, 100112 (2021).

34. Liao, P. S., Chen, T. S. & Chung, P. C. A fast algorithm for multilevel thresholding. *J. Inf. Sci. Eng.* **17**, 713–727 (2001).

35. Schindelin, J. *et al.* Fiji: An open-source platform for biological-image analysis. *Nat. Methods* **9**, 676–682 (2012).

36. Lowe, D. G. Distinctive image features from scale-invariant keypoints. *Int. J. Comput. Vis.* **60**, 91–110 (2004).

37. Demers, H., Poirier-Demers, N., de Jonge, N. & Drouin, D. Three-Dimensional Electron Microscopy Simulation with the CASINO Monte Carlo Software. *Microsc. Microanal.* **17**, 612–613 (2011).

38. Salvat, F., Jablonski, A. & Powell, C. J. Elsepa - Dirac partial-wave calculation of elastic scattering of electrons and positrons by atoms, positive ions and molecules. *Comput. Phys. Commun.* **165**, 157–190 (2005).

39. Joy, D. C. & Luo, S. An empirical stopping power relationship for low-energy electrons. *Scanning* **11**, 176–180 (1989).

40. OpenAI, 2023. ChatGPT-4 [AI language model]. https://www.openai.com/chatgpt (2023).

41. Landesfeind, J., Pritzl, D. & Gasteiger, H. A. An Analysis Protocol for Three-Electrode Li-Ion Battery Impedance Spectra: Part I. Analysis of a High-Voltage Positive Electrode. *J. Electrochem. Soc.* **164**, A1773–A1783 (2017).

42. Landesfeind, J., Hattendorff, J., Ehrl, A., Wall, W. A. & Gasteiger, H. A. Tortuosity Determination of Battery Electrodes and Separators by Impedance Spectroscopy. *J. Electrochem. Soc.* **163**, A1373–A1387 (2016).
